# Synthesis and Biological Evaluation of ^99m^Tc(I) Tricarbonyl Complexes Dual-Targeted at Tumoral Mitochondria

**DOI:** 10.3390/molecules26020441

**Published:** 2021-01-15

**Authors:** Diogo Figueiredo, Célia Fernandes, Francisco Silva, Elisa Palma, Paula Raposinho, Ana Belchior, Pedro Vaz, António Paulo

**Affiliations:** 1 C2TN Centro de Ciências e Tecnologias Nucleares, Instituto Superior Técnico, Universidade de Lisboa, Estrada Nacional 10 (km 139,7), 2695-066 Bobadela LRS, Portugal; diogoloureiro@ctn.tecnico.ulisboa.pt (D.F.); fsilva@ctn.tecnico.ulisboa.pt (F.S.); elisa@ctn.tecnico.ulisboa.pt (E.P.); paular@ctn.tecnico.ulisboa.pt (P.R.); anabelchior@tecnico.ulisboa.pt (A.B.); pedrovaz@ctn.tecnico.ulisboa.pt (P.V.); 2 DECN—Departamento de Engenharia e Ciências Nucleares, Instituto Superior Técnico, Universidade de Lisboa, Estrada Nacional 10 (km 139,7), 2695-066 Bobadela LRS, Portugal

**Keywords:** radiopharmaceuticals, auger emitters, technetium-99m, targeted radionuclide therapy (TRT), mitochondria targeting

## Abstract

For effective Auger therapy of cancer, the Auger-electron emitters must be delivered to the tumor cells in close proximity to a radiosensitive cellular target. Nuclear DNA is considered the most relevant target of Auger electrons to have augmented radiotoxic effects and significant cell death. However, there is a growing body of evidence that other targets, such as the mitochondria, could be relevant subcellular targets in Auger therapy. Thus, we developed dual-targeted ^99m^Tc(I) tricarbonyl complexes containing a triphenylphosphonium (TPP) moiety to promote accumulation of ^99m^Tc in the mitochondria, and a bombesin peptide to provide specificity towards the gastrin releasing peptide receptor (GRPr) overexpressed in prostate cancer cells. The designed dual-targeted complex, **^99m^Tc-TPP-BBN**, is efficiently internalized by human prostate cancer PC3 cells through a specific GRPr-mediated mechanism of uptake. Moreover, the radioconjugate provided an augmented accumulation of ^99m^Tc in the mitochondria of the target tumor cells, most probably following its intracellular cleavage by cathepsin B. In addition, **^99m^Tc-TPP-BBN** showed an enhanced ability to reduce the survival of PC3 cells, in a dose-dependent manner.

## 1. Introduction

Nuclear medicine can play a pivotal role in precision diagnosis and treatment of cancer due to the unique features of nuclear imaging technologies (Positron Emission Tomography (PET) and Single Photon Emission Computed Tomography (SPECT)), increasing the availability of emerging medical radionuclides and development of innovative radiopharmaceuticals. In this context, the last decade has seen a rapid growth in the use of theranostic radionuclides for the treatment and imaging of cancers, namely for targeted radionuclide therapy (TRT) of prostate cancer and neuroendocrine tumors [1,2]. Among the available theranostic radionuclides, Auger emitters (AE) have very attractive properties for TRT of cancer since the nanometer-micrometer range of Auger electrons results in high linear energy transfer (LET) with potential lethal damage in cancer cells. Moreover, many of them are commercially available and are already used clinically in nuclear medicine imaging (e.g., ^67^Ga, ^99m^Tc or ^111^In) [3]. In addition, TRT using AE is usually considered with greater potential to treat small size cancers and metastases, because of their high level of cytotoxicity, high LET, and short range biological effectiveness [4]. The short range of Auger electrons also favors selective therapies with reduction of side-effects. ^99m^Tc is not an ideal AE for TRT due to its relatively low Auger electron yield with emission of an average of four Auger electrons and ≈0.11 internal conversion electrons per decay [3]. However, it can be considered a readily available “model” radionuclide obtained in situ at economical prices through the ^99^Mo/^99m^Tc generator, useful to validate the design of new classes of Auger-electron emitting radioconjugates.

The highest relative biological effectiveness (RBE) of AE results when these radionuclides are internalized by highly radiosensitive organelles, namely the cell nucleus. Therefore, the targeting of these organelles in specific cancer cells is expected to enhance therapeutic effects at lower administrated doses. Towards this goal, several research groups have studied radiolabeled DNA intercalators to promote accumulation of AE in the cell nucleus in closer proximity with DNA [3]. For this purpose, we studied ^99m^Tc(I) tricarbonyl complexes carrying acridine orange (AO) derivatives as DNA intercalating groups [5,6,7]. Our results with the ^99m^Tc(I)-AO complexes have shown that ^99m^Tc can cause significant radiation-induced DNA damage, both in vitro in plasmid models and in vivo in human prostate cancer PC3 cells [6,7]. Noticeably, we have proved that there is a marked dependence of the biological effectiveness of ^99m^Tc Auger electrons on the ^99m^Tc-DNA distance, even for a relatively small increase of such distance (e.g., 10.80 Å vs. 12.92 Å), as estimated by molecular modelling simulations of the DNA-intercalated complexes [6]. 

Although the current paradigm in radiobiology posits that nuclear DNA is the primary target for ionizing radiation, there is a growing body of evidence that other sensitive sites besides the nuclear DNA, such as the mitochondria, could be critical targets in TRT [8]. Mitochondria are very complex organelles that harbor their own DNA and a double membrane structure with unusual lipid composition and a high membrane potential of 150–180 mV (negative inside) [9]. Mutations of mitochondrial DNA (mtDNA) are associated with multiple disorders, ranging from neurodegenerative diseases to cancers [10]. Cancer cells exhibit mitochondrial dysfunction, genetic instability with alterations, and highly glycolytic activities [11]. The mitochondria have emerged as an interesting but relatively understudied extranuclear target of ionizing radiation. Circular mitochondrial DNA, like genomic DNA, is sensitive to ionizing radiation-induced damage. In addition, some investigators have suggested that ionizing radiation can alter mitochondrial function, induce mitochondrial oxidative stress, and cause mitochondrial-induced apoptosis [12].

Surprisingly, the studies of mitochondria-targeted radioconjugates for improved TRT of cancer remain scarce. A rare example is the elegant study by Yu et al. with mitochondria-targeting ^177^Lu-porphyrin-PEG nanocomplexes (PEG = polyethylene glycol). These nanoconstructs, containing the β^−^ emitter ^177^Lu and a photosensitizer, caused an increase in reactive oxygen species (ROS) and a reduction in cell viability, in particular when combined with photodynamic therapy [13]. By contrast, a relatively large number of imaging studies have been reported with mitochondria-tropic radioprobes, mainly for in vivo tumor detection. This included several lipophilic cations originally developed for myocardial perfusion SPECT imaging, such as the cardiac agent ^99m^Tc-MIBI (^99m^Tc-Sestamibi) or the ^99m^Tc-TMEOP complex introduced by our group [14,15,16,17,18]. Among the mitochondrial-targeting moieties, the most widely applied is triphenylphosphonium (TPP), which is a delocalized cationic lipid that readily penetrates through the mitochondrial membrane due to the highly negative mitochondrial membrane potential [11]. Taking advantage of their mitotropic properties, several research groups have studied TPP derivatives in the design of radioprobes for in vivo targeting of mitochondria. 

The majority of radiolabeled TPP-derivatives have been obtained using PET radionuclides, like ^64^Cu and ^18^F [19,20,21,22,23,24,25]. In the past several years, many efforts have also been made to obtain ^99m^Tc-labeled TPP derivatives for mitochondria targeting and SPECT imaging of cancer. Zhao’s group developed a mercaptoacetyltriglycine (MAG3) Tc(V) oxocomplex bearing a TPP derivative for the early detection of breast tumors, but the neutral charge of the complex appeared to be a disadvantage for the desired purpose [26]. Our group and other authors have studied several TPP-containing ^99m^Tc(I) tricarbonyl complexes as radioactive metalloprobes targeted to the mitochondria for in vivo detection of tumor tissues [27,28,29]. So far, the tested radiolabeled TPP-derivatives presented serious limitations due to low tumor specificity and/or high undesirable buildup in the chest and abdomen, which should lead to undesired side-effects like cardiotoxicity within a radiotherapeutic approach.

To improve the cancer specificity of radiolabeled TPP-derivatives targeted at the mitochondria, we have considered multifunctional ^99m^Tc(I) tricarbonyl complexes containing the TPP moiety and a bombesin (BBN) peptide derivative that specifically recognizes the gastrin releasing peptide receptor (GRPr) overexpressed in prostate cancer cells. The idea of such radioconjugates was inspired by our previous results with a pyrazolyl-diamine ^99m^Tc(I) complex (Tc-AO-BBN) carrying a bombesin sequence (BBN[7-14]) for GRPr targeting and an AO group for DNA intercalation. The Tc-AO-BBN complex presented specific cell targeting with a pronounced nuclear internalization in prostate cancer PC3 cells. This result showed for the first time that multifunctional complexes can transport an Auger-emitting radiometal, in a cell-specific way, to the nucleus of tumoral cells [5]. Most relevantly, the presence of a triglycine (Gly-Gly-Gly) linker between the BBN[7-14] sequence and the pyrazolyl-diamine chelator had a determinant influence on the nuclear internalization of the radioconjugate, which was almost negligible when a Ser-Gly-Ser linker was used instead of Gly-Gly-Gly. Most probably, Gly-Gly-Gly acts as a cathepsin B cleavable linker that leads to the release of an AO-containing ^99m^Tc(I) fragment inside the cells with better ability to reach the cell nucleus. In fact, the GlyGlyGly sequence has been already successfully applied as a cathepsin B cleavable linker to optimize the drug delivery properties of antibody-drug conjugates, in the same way as more popular linkers containing Val-Ala and Val-Cit motifs [30,31].

**Figure 1:**
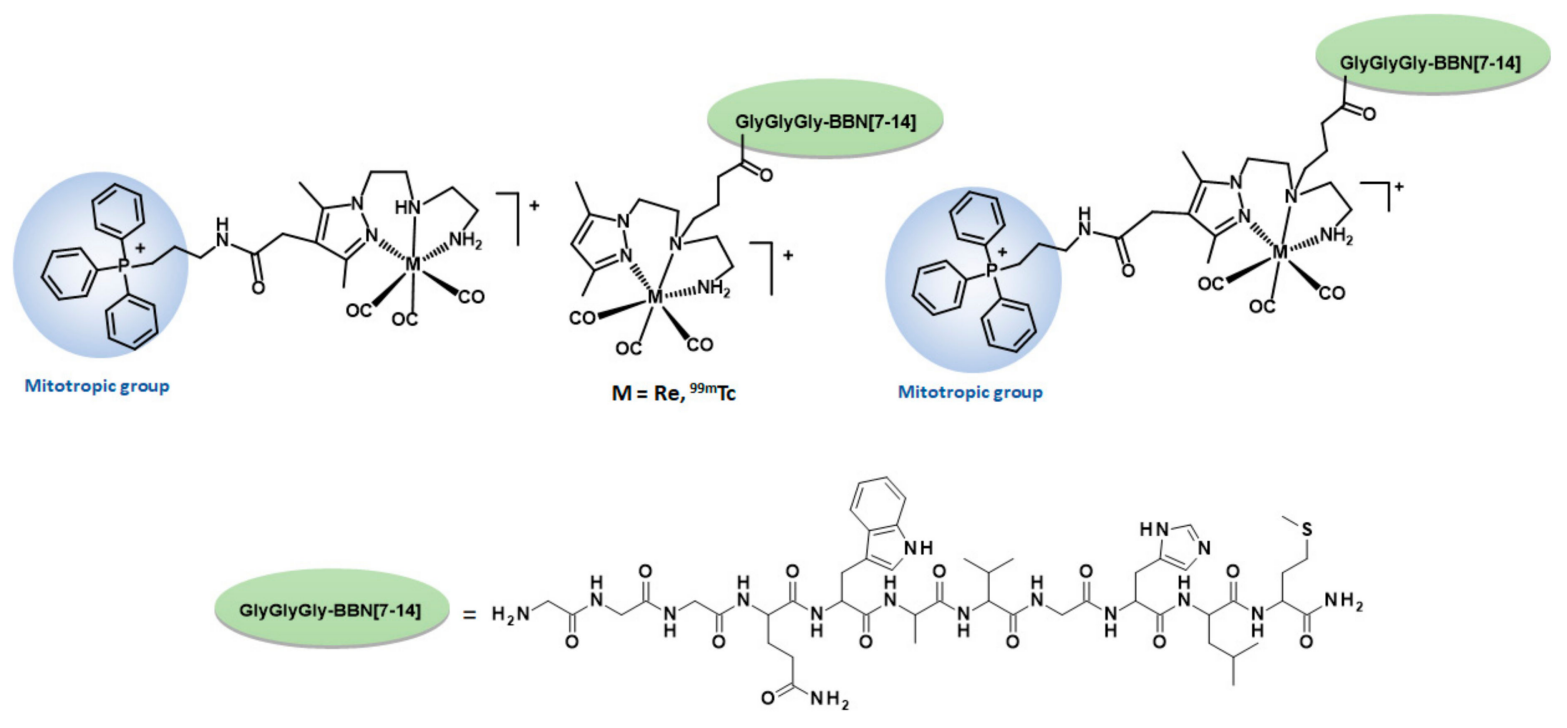
Chemical structures of the ^99m^Tc/Re complexes studied, bearing a mitotropic group and/or a BBN derivative targeting the GRPr.

In this work, we describe a new multifunctional ^99m^Tc(I) tricarbonyl complex (**Tc-TPP-BBN**) containing the mitochondria-targeting TPP moiety and a BBN[7-4] sequence attached to the pendant arm of a pirazolyl-diamine chelator through a Gly-Gly-Gly linker (Figure 1). We hypothesized that this multifunctional radioconjugate will be recognized firstly by the extracellular target (GRPr) that will trigger its cell internalization by endocytosis. Once inside the cell, the radioconjugate should be cleaved by cathepsin B at the Gly-Gly-Gly linker to release a smaller-sized ^99m^Tc-labeled TPP moiety with better ability to interact with its intracellular target (mitochondria) for an enhanced mitochondrial accumulation of the radioactive cargo. To evaluate the possibility of such dual targeting, the cellular internalization and mitochondria uptake of **^99m^Tc-TPP-BBN** in GRPr-positive PC-3 cells were compared with those of congener ^99m^Tc complexes without the peptide sequence (**^99m^Tc-TPP**) and without the TPP moiety (**^99m^Tc-BBN**) (Figure 1). One of our goals was to assess how the mitochondrial uptake of ^99m^Tc will influence the radiotoxic effects induced by this Auger emitter in human prostate cancer PC3 cells. To clarify this aspect, the survival of PC3 cells exposed to increasing activities of the radiocomplexes **^99m^Tc-TPP-BBN** and **^99m^Tc-BBN** was studied using the clonogenic assay, as also reported herein. Finally, the cleavage of the Gly-Gly-Gly linker by cathepsin B was studied using in vitro enzymatic assays and the complex **Re-BBN** (Section 2.3.1). Rhenium complexes are commonly used as non-radioactive surrogates of ^99m^Tc congeners benefiting from the physico-chemical similarities of these two elements that tend to form isostructural complexes with similar biological properties [32].

## 2. Results

### 2.1. Chemistry

As described below, we have synthesized single targeted TPP derivatives (**TPP-pz** and **M-TPP** (M = Re, ^99m^Tc)) and BBN derivatives carrying or not the TPP moiety (**M-TPP-BBN** and **M-BBN** (M = Re, ^99m^Tc)) (Figure 1). The pyrazole-diamine bifunctional chelator was selected to stabilize the Re(I)/^99m^Tc(I) tricarbonyl complexes taking into consideration the excellent coordination capability of this type of chelator towards the *fac*-[M(CO)_3_]^+^ (M = Re, Tc) moiety, forming complexes with high in vitro and in vivo stability [32,33,34]. Most importantly, this class of chelators allows a versatile functionalization that facilitated the synthesis of the dual-targeted compounds carrying a BBN derivative and a mitotropic TPP moiety. For this purpose, the 4-position of the pyrazole ring was used in this study to introduce the TPP derivative and a pendant butyric arm was attached at the secondary amine of the ligand backbone for conjugation of a BBN derivative containing a triglycine cleavable linker.

#### 2.1.1. Synthesis and Characterization of Single-Targeted TPP Derivatives: Chelators and Re Complexes

**Scheme 1:**
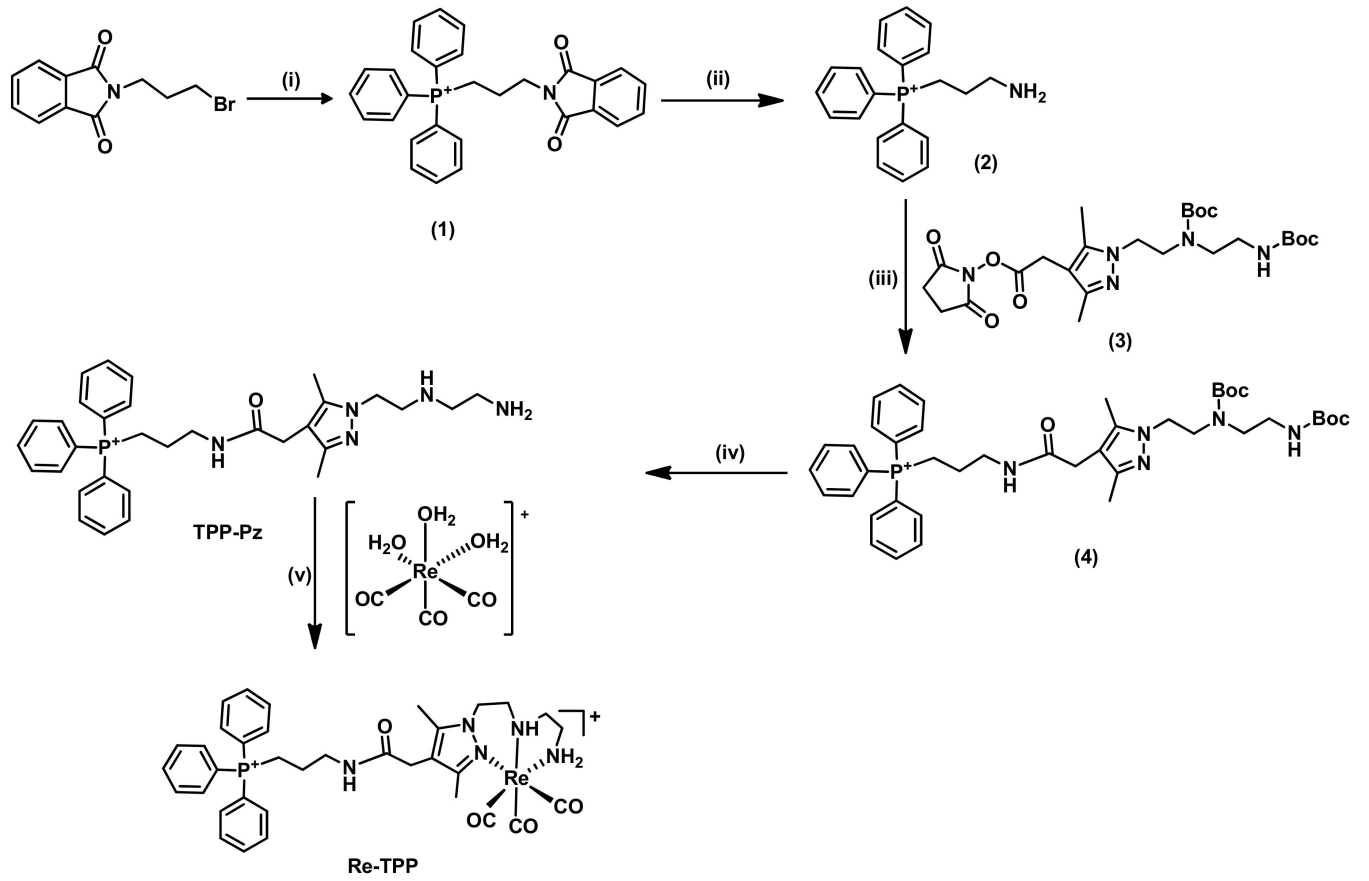
Synthetic route towards the ligand **TPP-Pz** and the rhenium complex **Re-TPP**. (i)Triphenylphosphine (PPh_3_), CH_3_CN, reflux; (ii) Hydrazine hydrate 50–60%, ethanol, reflux; (iii) DIPEA, CH_2_Cl_2_, RT; (iv) TFA, CH_2_Cl_2_, RT; (v) methanol, reflux.

A TPP derivative bearing a free amine with a three-carbon spacer (compound **2**) was used to introduce the TPP moiety in the 4-position of the pyrazolyl ring of the chelator framework. Compound **2** was obtained using bromopropyl phthalimide as the starting alkylating agent (Scheme 1). The conjugation of the phthalimide precursor with triphenylphosphine (PPh_3_) was performed in acetonitrile at reflux for 24 h to ensure complete reaction. The resulting product, compound **1**, was isolated with high yield (92%) after purification by silica gel column chromatography. The next step was the phthalimide deprotection using a large excess of hydrazine at reflux in ethanol. To release the free amine (as a salt), concentrated HCl was added and the byproduct phthalhydrazide was removed by filtration. Then, compound **2** was extracted to chloroform after basification with NaOH. The TPP derivative **2** was obtained with 84% yield and was fully characterized by multinuclear NMR spectroscopy (^1^H, ^13^C and ^31^P-NMR) and ESI-MS analysis. The obtained analytical data are consistent with the molecular structure of **2**. In particular, the ^31^P-NMR spectrum exhibited only one signal (23.6 ppm) confirming the presence of a single phosphorous-containing species without oxidation of the TPP group.

The incorporation of this TPP derivative **2** at the 4-position of the pyrazolyl ring was done using a BOC-protected pyrazolyl-diamine chelator **3** functionalized with a activated NHS ester at that 4-position, which was synthesized as previously described by us [35]. Compound **3** was reacted with **2** in DCM in the presence of diisopropylethylamine (DIPEA) as base. The resulting BOC-protected compound **4** was recovered as a pale yellow oil and its identity confirmed by ESI-MS analysis with the detection of a molecular ion at *m*/*z* = 742.8 [M]^+^ (*m*/*z* calcd for [C_42_H_57_N_5_O_5_P]^+^: 742.41). Consistently, the ^1^H-NMR spectrum of **4** shows signals attributable to the aromatic protons of the triphenylphosphine moiety and a unique signal at 24.39 ppm in its ^31^P-NMR spectrum, slightly downfield shifted relatively to compound **2**. Removal of the Boc-protecting groups of **4** with TFA in DCM (1/1) afforded the ligand **TPP-Pz** in quantitative yield, which was isolated as a pale-yellow oil. Further purification of **TPP-Pz** was performed by solid phase extraction using Sep-Pak C_18_ cartridges before its full characterization and use on the synthesis of its Re complex (**Re-TPP**) (Scheme 1).

Complex **Re-TPP** was synthesized by reaction of the aquo-tricarbonyl precursor [Re(CO)_3_(H_2_O)_3_]Br with an equimolar amount of the **TPP-Pz** chelator in refluxing methanol. The purification of **Re-TPP** was achieved by RP-HPLC and its characterization comprised multinuclear NMR spectroscopy (^1^H, ^13^C and ^31^P-NMR) and ESI-MS analysis. The ^1^H NMR spectrum of **Re-TPP** shows a series of multiplet signals in the range 2.0–5.0 ppm that are due to the diastereotopic CH_2_ protons from the chelator framework directly involved in the coordination to the metal and three broad resonances for the respective N-H protons (3.86, 5.41, and 6.91 ppm), as typically observed for Re(I) tricarbonyl complexes with pyrazolyl-diamine chelators [27,35,36]. The ^31^P NMR spectrum displays a unique signal at 23.05 ppm that appears at a slightly higher field than in the respective **TPP-Pz** chelator (23.58 ppm), showing that the coordination of **TPP-Pz** to the metal centre was not accompanied by oxidation of the TPP group or other degradation process. The complex **Re-TPP** has been used as a non-radioactive surrogate of the congener ^99m^Tc complex (**Tc-TPP**) to assess its chemical identity by means of HPLC comparison, which is a common and well accepted practice in radiopharmaceutical chemistry due to the physico-chemical similarities of Re and Tc complexes.

#### 2.1.2. Synthesis and Characterization of BBN Derivatives: Peptides, Chelators, and Re Complexes

**Scheme 2:**
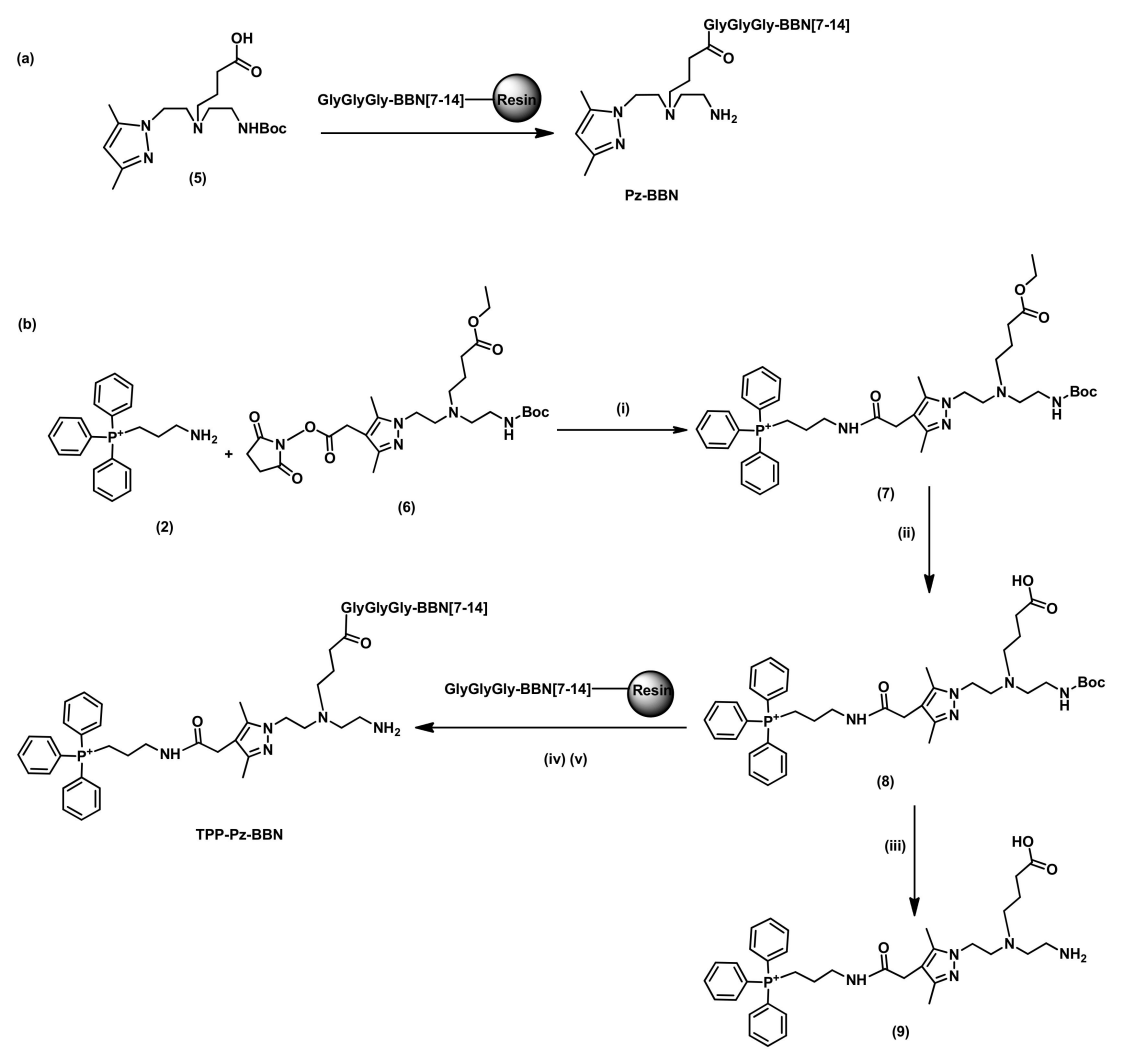
(**a**) Synthetic route towards **Pz-BBN**; (**b**) synthetic route towards the dual-targeted ligand **TPP-Pz-BBN**. (i) DIPEA, DMF, RT; (ii) NaOH (aq), 50–60 °C; (iii) TFA, CH_2_Cl_2_, RT; (iv) HBTU, DIPEA, DMF, RT; (v) TFA:TIS:H_2_O.

The synthesis of the several BBN derivatives (pyrazolyl-diamine chelators and their Re complexes) was performed based on solid phase peptide synthesis (SPPS) methodologies and starting from the G3BBN[7-14] peptide containing a triglycine linker, using the appropriate pyrazolyl-diamine precursors and procedures similar to those described previously to obtain the **Pz-BBN** conjugate (Scheme 2a) [37].

To obtain the dual-targeted bifunctional chelator (**TPP-Pz-BBN**), carrying a TPP moiety and the G3BBN[7-14] sequence, we synthesized a pyrazolyl-diamine derivative (compound **8**) functionalized with the TPP moiety at the 4-position of the pyrazolyl ring and containing a butyrate arm at the central amine for coupling of the BBN peptide (Scheme 2(b)). As a first step, the activated ester **6** was synthesized as previously reported and reacted with the free amine of compound **2** in dry DMF and in the presence of DIPEA [5]. The reaction afforded the ethyl ester derivative **7** containing the TPP moiety, which was recovered in good yield (70%) after purification by column chromatography using DCM/MeOH as eluent. The formation of compound **7** was supported by the presence of the aromatic protons of the TPP moiety in the ^1^H-NMR spectrum and was confirmed by the mass spectrometry analysis (*m*/*z* calcd for [C_43_H_59_N_5_O_5_P]^+^: 756.42, found: 756.5 [M]^+^, 378.9 [M + H]^2+^). In the next step, the removal of the ethyl protecting group was performed by selective hydrolysis with sodium hydroxide in aqueous solution, under overnight reflux according to described methodology [5]. However, these harsh conditions led to the oxidation of the triphenylphosphonium group, as indicated by ^1^H and ^31^P-NMR analysis of the isolated product and confirmed by ESI-MS analysis (Figures S1–S3). To overcome this drawback, the reaction was studied under softer conditions using less concentrated NaOH and lower temperature. The reaction progress was monitored by HPLC which showed a complete removal of the ethyl ester in only 2 h at 50–60 °C. Under these conditions, the purified Boc-protected compound **8** was obtained in high yield (92%). Compound **8** has been characterized by ^1^H- and ^31^P-NMR and ESI-MS, which confirmed the formation of the desired compound without oxidation of the phosphonium group. Removal of the Boc-protecting group of **8** by TFA afforded compound **9** that after purification by Sep-Pak C18 was used in the synthesis of the dual-targeted Re complex (**Re-TPP-BBN**).

The G3BBN[7-14] peptide was synthesized by a standard Fmoc strategy. Amino acids were coupled to a Rink amide resin (MBHA) and at the end of the synthesis, the final Fmoc group was removed to yield the resin-bound G3BBN[7-14] with a free terminal amine. The Boc-protected chelator **8** was conjugated to the terminal free amine of the resin bound G3BBN[7-14] peptide using solid-phase synthesis methodologies and the activating agent 2-(1H-Benzotriazole-1-yl)-1,1,3,3-tetramethyluronium hexafluorophosphate (HBTU) in the presence of DIPEA (Scheme 2b). As detailed in the “Materials and Methods” section, the resulting dual-targeted chelator **TPP-Pz-BBN** was cleaved from the resin by a cleavage cocktail, concentrated and precipitated with cold diethyl ether. Thereafter, it was purified by RP-HPLC using a gradient elution of water/acetonitrile containing 0.1% of trifluoroacetic acid (TFA) and obtained with a high purity degree (>95%). The ESI-MS analysis of **TPP-Pz-BBN** acquired in positive mode detected the molecular ion [M]^+^ at *m*/*z* 1720.9 and the protonated ion [M + H]^2+^ at *m*/*z* 861.7 with isotope distribution patterns matching well the theoretical ones and validating the assigned structure (Figure S7). **TPP-Pz-BBN** has been used to obtain the **^99m^Tc-TPP-BBN** complex by direct reaction with the [^99m^Tc(CO)_3_(H_2_O)_3_]^+^ precursor, as described below in the “Radiochemistry” section.

**Scheme 3:**
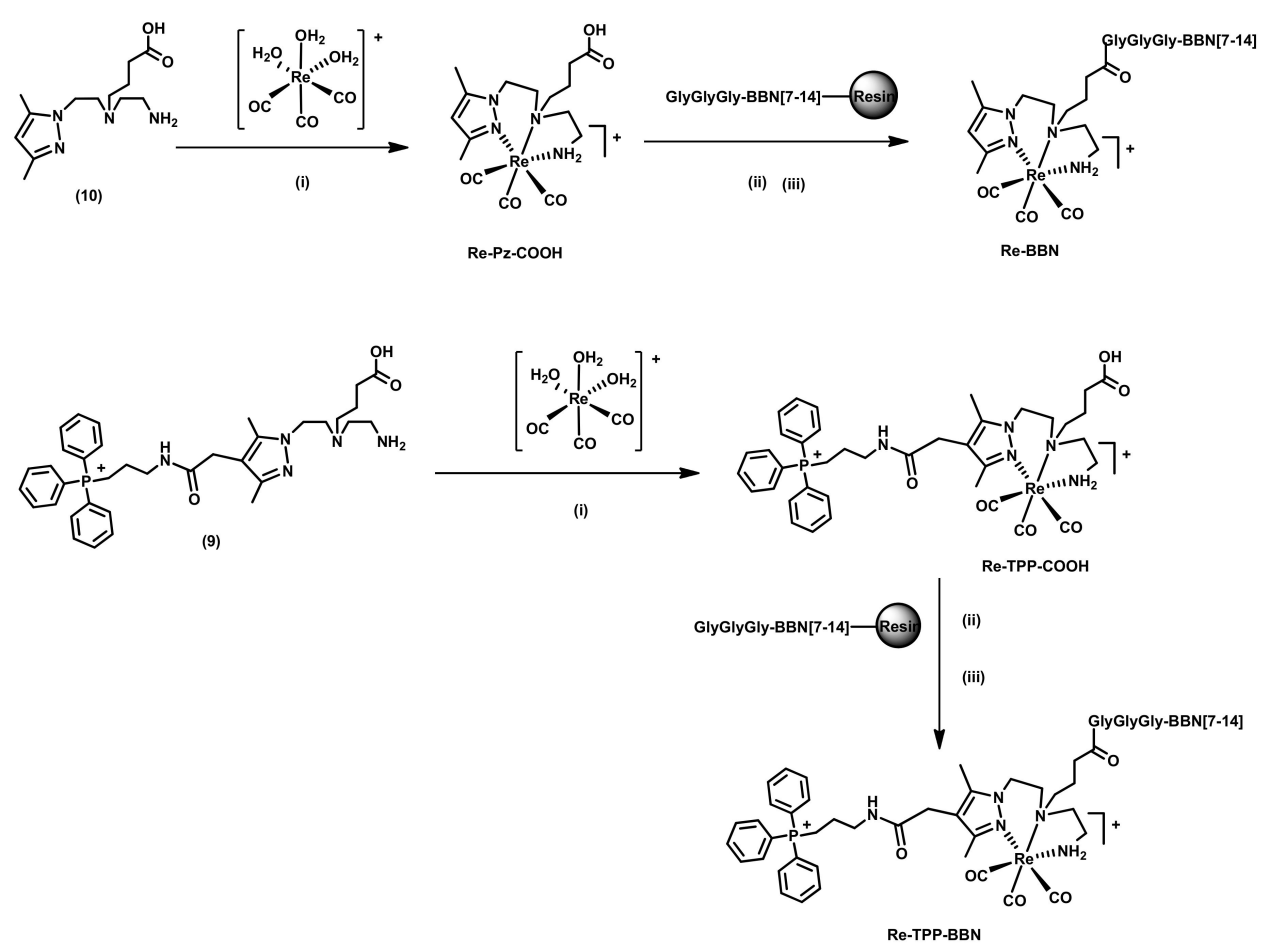
Synthesis of Re complexes (with and without TPP) bearing the G3BBN[7-14] sequence. (i) Methanol, reflux; (ii) HBTU, DIPEA, DMF, RT; (iii) TFA:TIS:H_2_O.

Unlike the ^99m^Tc congeners, the rhenium-peptide conjugates **Re-BBN** and **Re-TPP-BBN** were not synthesized by direct complexation reactions with the corresponding bifunctional chelators **Pz-BBN** and **TPP-Pz-BBN**, respectively. Instead, these rhenium conjugates were synthesized by solid-phase reactions between resin bound G3BBN[7-14] and complexes **Re-Pz-COOH** and **Re-TPP-COOH** (Scheme 3), as described above for **TPP-Pz-BBN**. After HPLC purification, **Re-BBN** and **Re-TPP-BBN** were analyzed by ESI-MS. The ESI(+) mass spectra confirmed the formation of the desired conjugates being observed the respective molecular ions, which appeared at *m*/*z* 996.0 ([M]^2+^) and 665.9 ([M + H]^3+^) in the case of **Re-TPP-BBN** (Figure S8). As described below, **Re-BBN** was used as a model compound in the enzymatic assays with cathepsin B to evaluate the possible cleavage of the conjugates at the Gly-Gly-Gly linker and **Re-TPP-BBN** was used as a surrogate of the congener ^99m^Tc-radiopeptide.

### 2.2. Radiochemistry

#### 2.2.1. Synthesis of ^99m^Tc(I) Complexes

**Scheme 4:**
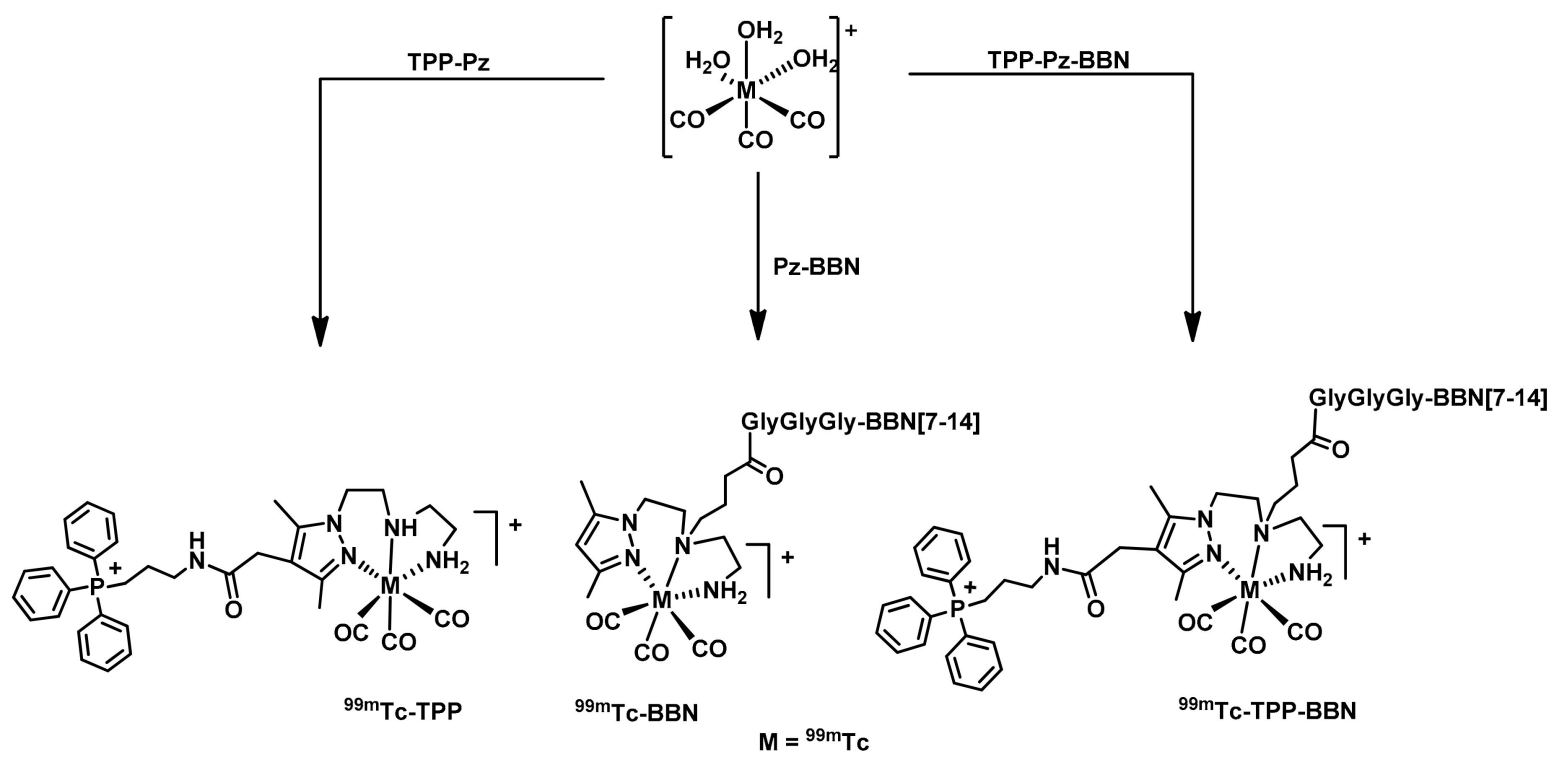
Synthesis of ^99m^Tc complexes: **^99m^Tc -TPP**; **^99m^Tc-BBN**; **^99m^Tc-TPP-BBN**. (Aqueous solution pH = 7, 30 min, 100 °C; [TPP-Pz] = 5 × 10^−5^ M; [Pz-BBN] = 5 × 10^−5^ M; [TPP-Pz-BBN] = 10^−4^ M).

**Figure 2:**
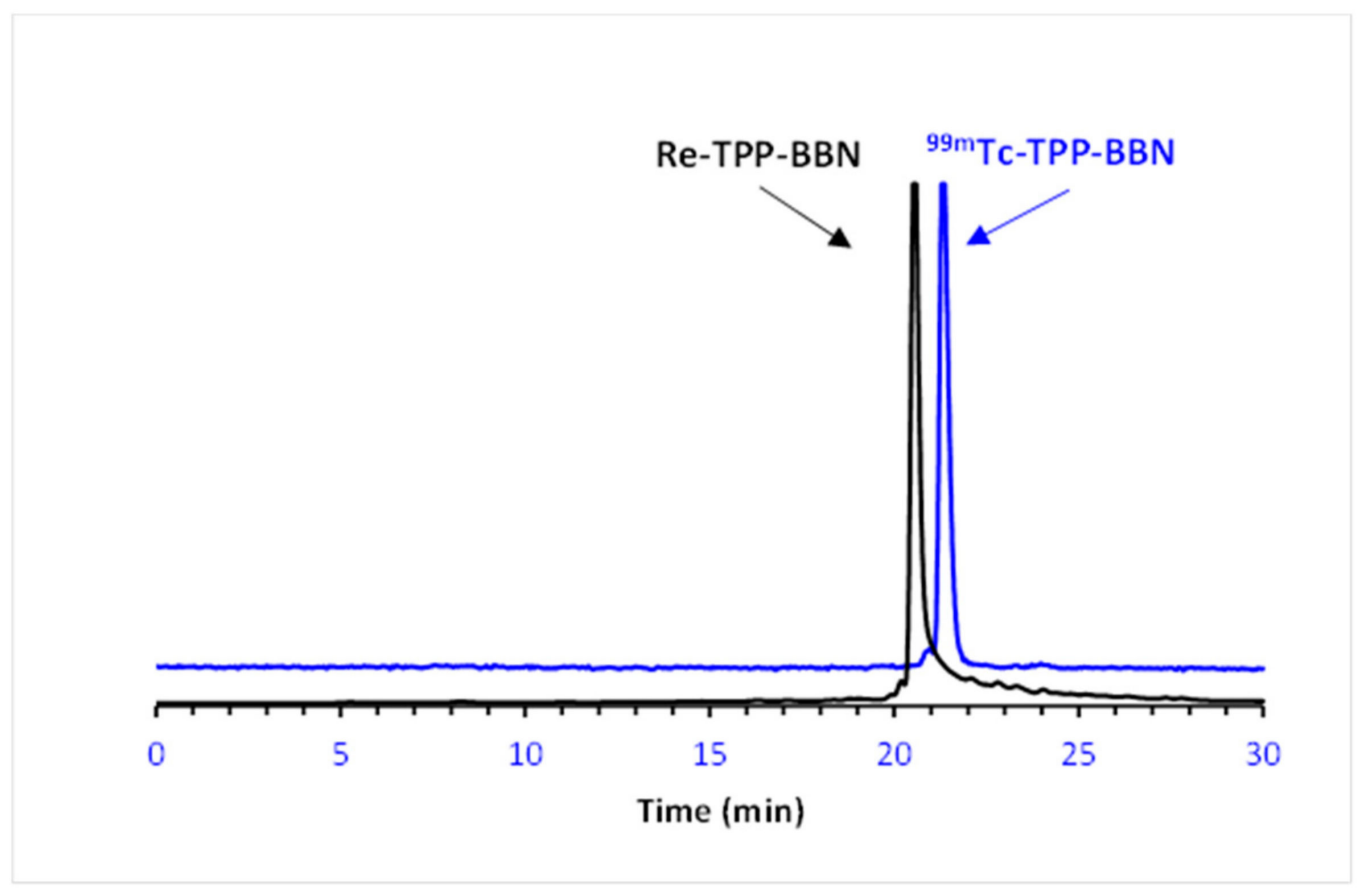
HPLC chromatograms of co-injected rhenium complex (**Re-TPP-BBN**) (UV detection; 254 nm) and ^99m^Tc complex (**^99m^Tc-TPP-BBN**)(γ detection; HPLC—method 2).

The ^99m^Tc(I) tricarbonyl complexes (**^99m^Tc-BBN**, **^99m^Tc-TPP** and **^99m^Tc-TPP-BBN**) were obtained in aqueous solution by ligand-exchange reaction of the radioactive precursor *fac*-[^99m^Tc(H_2_O)_3_(CO)_3_]^+^ with the corresponding bifunctional ligands (**Pz-BBN**, **TPP-Pz**, **TPP-Pz-BBN**), at 100 °C for 30 min (Scheme 4). **^99m^Tc-TPP** was obtained in almost quantitative radiochemical yield (>95%) using ligand concentrations as low as 5 × 10^−5^ M. In contrast, **^99m^Tc-BBN** and **^99m^Tc-TPP-BBN** were obtained with a slightly lower yield (90%) due to the formation of a radiochemical impurity that is most probably related with the oxidation of the methionine residue. Nevertheless, this radiochemical impurity was efficiently removed by HPLC purification of the BBN-containing radiocomplexes. All the ^99m^Tc complexes were purified by HPLC to remove the excess of the corresponding ligand leading to complexes with high radiochemical purity (RCP > 95%) and high specific activity. The specific activity of the purified radioconjugates can be considered the same as that of ^99m^Tc obtained from a ^99^Mo/^99m^Tc generator that has been submitted to a 24 h prior elution, i.e., ca. 6.4 GBq/μmol. The presence of the chelators carrying the BBN and TPP derivatives could lead to potential interference in the cellular studies of the ^99m^Tc complexes described below, namely blockade of the GRPr or cytotoxic action affecting the survival rate of the cells. The chemical identity of **^99m^Tc-BBN**, **^99m^Tc-TPP**, and **^99m^Tc-TPP-BBN** has been ascertained by comparing their analytical RP-HPLC radiochromatograms with UV-vis traces of the corresponding rhenium congeners, as exemplified for **^99m^Tc-TPP-BBN**/**Re-TPP-BBN** in Figure 2.

#### 2.2.2. In Vitro Stability and Lipophilicity of the ^99m^Tc(I) Complexes

The in vitro evaluation of ^99m^Tc complexes included the study of their lipophilicity and stability under physiological conditions and in cell culture medium.

The stability of the radioactive complexes was evaluated upon incubation of the complexes with phosphate-buffered saline (PBS) pH 7.4 and cell culture medium (DMEM), at 37 °C. Analysis of the samples by RP-HPLC showed that **^99m^Tc-TPP** (Figures S13 and S14) is stable up to 24 h, as no decomposition products or reoxidation to pertechnetate was detected. On the other hand, the complexes **^99m^Tc-BBN** (Figures S10 and S11) and **^99m^Tc-TPP-BBN** (Figures S16 and S17) exhibited a teeny tendency to undergo oxidation at the methionine residue (10% oxidized form after 24 h incubation in PBS).

The lipophilicity can play a decisive role in the pharmacokinetics and pharmacodynamics of a drug substance affecting its ability to cross membranes by passive diffusion and reach target tissues. This should be particularly relevant for the **^99m^Tc-TPP** complex due to the absence of the targeting BBN peptide. The lipophilicity of the ^99m^Tc complexes was assessed based on the determination of the n-octanol/0.1 M PBS pH 7.4 partition coefficients by the shake-flask method. The following Log P_o/w_ values were found: Log P_o/w_ = −0.75 (^99m^Tc-BBN); Log P_o/w_ = 0.52 (^99m^Tc-TPP); Log P_o/w_ = 0.45 (^99m^Tc-TPP-BBN). **^99m^Tc-BBN** is highly hydrophilic and the dicationic **^99m^Tc-TPP** is the most lipophilic complex, which certainly reflects the presence of the BBN peptide and phenyl groups, respectively. The dual-targeted complex **^99m^Tc-TPP-BBN** presented an intermediate lipophilicity due to the contribution of both targeting moieties for its hydro/lipophilicity, the lipophilic TPP group, and the hydrophilic G3BBN sequence.

### 2.3. Biological Studies

#### 2.3.1. In Vitro Enzymatic Assays

One crucial issue in the design of the dual-targeted complex presented in this study, aimed at the targeting of the mitochondria of GRPr-positive prostate cancer cells, was the presence of a triglycine linker between the BBN peptide and the organometallic fragment carrying the mitotropic group. The intracellular enzymatic cleavage of this linker should release the peptide fragment producing a smaller-sized complex carrying the TPP group with enhanced ability to accumulate in the mitochondria of tumor cells.

We have considered that the presence of the TPP group in **Re-TPP-BBN**, far away from the triglycine linker, would not affect the ability of the enzyme to recognize the substrate. Therefore, we have pursued the study of the cleavage of the Gly-Gly-Gly linker by cathepsin-B using the **Re-BBN** complex as a model. The enzymatic assays were performed using commercially available bovine cathepsin B and comprised the incubation of **Re-BBN** with a solution of the enzyme and monitoring of the metabolites formation over time by HPLC and ESI-MS analysis, according to procedures described in the literature [38].

**Figure 3:**
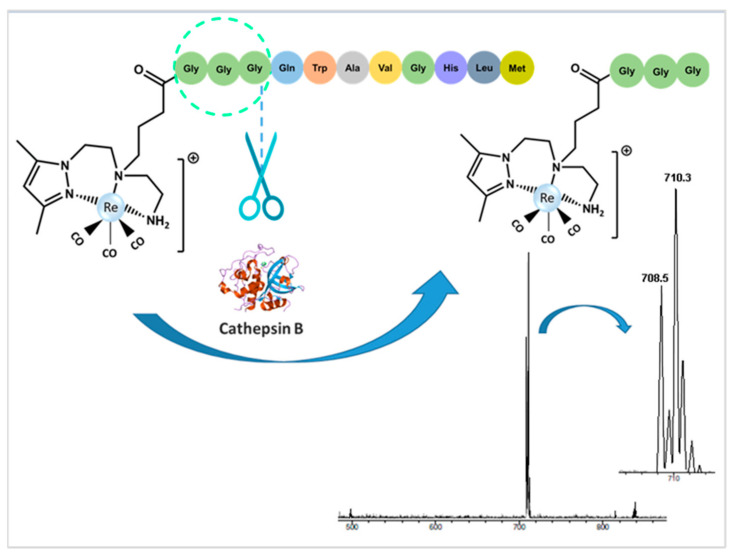
Schematic representation of the cleavage of the **Re-BBN** complex by cathepsin B and ESI-MS spectrum (positive mode) of the most predominant metabolite.

The reaction mixture was maintained with soft mixing at 37 °C and was analyzed overtime (2 to 48 h). After quenching the reaction, the products were separated by HPLC and analyzed by mass spectrometry. In the HPLC chromatograms, the increasing formation of metabolites over time was observed, while the amount of intact **Re-BBN** complex decreased (Figure S18). It is important to notice that the enzymatic cleavage at different positions of the BBN peptide affords different pairs of Re-containing fragments and peptide fragments, leading to a complex mixture of compounds. To identify the metabolites, the peaks observed in the HPLC chromatograms were collected and analyzed by ESI-MS. The most intense peak was identified as the metallated fragment resulting from the cleavage of the amide bond between the triglycine linker and the BBN[7-14], as proposed in Figure 3. The ESI-MS analysis in the positive mode detected the corresponding molecular ion [M]^+^ at *m*/*z* 708.5 with an isotope distribution pattern consistent with the proposed formulation, namely with the presence of one Re atom. These results confirmed that the triglycine linker in these organometallic complexes functionalized with the G3BBN[7-14] acts as a substrate of cathepsin B, as initially thought.

#### 2.3.2. Cellular Uptake and Internalization Studies

**Figure 4:**
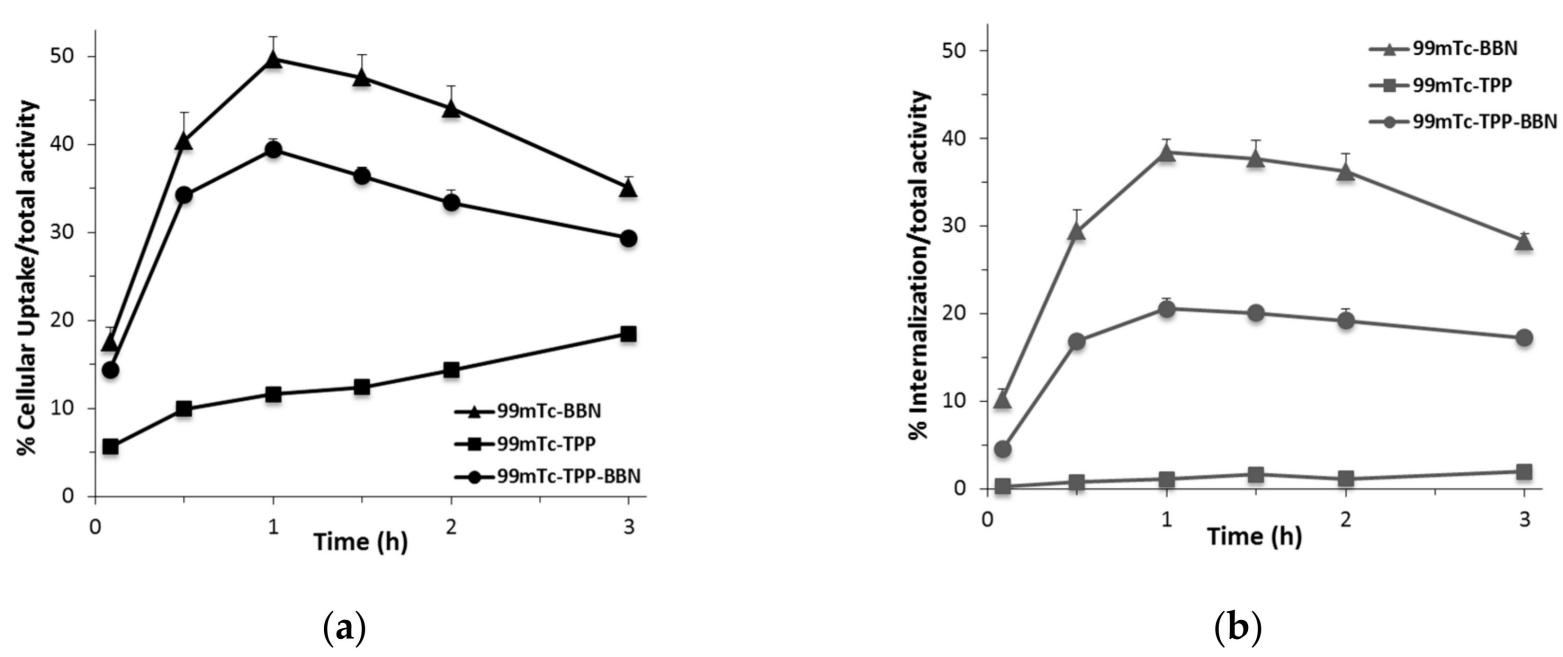
Time-dependent cellular uptake of ^99m^Tc-TPP-BBN (●), ^99m^Tc-TPP (■) and ^99m^Tc-BBN (▲) in PC3 cells at 37 °C. Cellular uptake (**a**) and internalization (**b**) were expressed as a percentage of the total (applied) activity. Results were calculated from independent biological replicates (*n* = 4) and are given as the average ± SEM.

In order to investigate the ability of the dual targeting complex **^99m^Tc-TPP-BBN** and related single-targeting complexes (**^99m^Tc-BBN** and **^99m^Tc-TPP**) to bind to PC3 cells, their cellular localization and distribution was evaluated by quantitative gamma-counting measurements. First, the cellular uptake and internalization of these radiocomplexes were evaluated in PC3 human prostate cancer cells at 37 °C, for up to 3 h, and results are represented in Figure 4. Surface-bound results are represented in Figure S19.

These studies revealed a time-dependent cellular uptake at 37 °C for all ^99m^Tc-complexes, with the BBN-bearing radiocomplexes binding faster and in a larger extent than **^99m^Tc-TPP** (Figure 4a). Indeed, the maximum values of the high cellular uptake of the BBN-containing radiocomplexes were reached at 1 h incubation time (49.7 ± 2.5% and 39.4 ± 1.2% for **^99m^Tc-BBN** and **^99m^Tc-TPP-BBN**, respectively), pinpointing the involvement of a receptor-mediated uptake. Furthermore, **^99m^Tc-BBN** and **^99m^Tc-TPP-BBN** displayed high levels of internalization with a kinetics similar to the cellular uptake kinetics (Figure 4b), in agreement with a GRP receptor specific mechanism In fact, at 1 h incubation, 38.4 ± 1.4% and 20.6 ± 1.2% of the applied **^99m^Tc-BBN** and **^99m^Tc-TPP-BBN** activities, respectively, were inside the cell. By contrast, **^99m^Tc-TPP** presented a negligible level of internalization (0.8% and 1.9% after 1 h and 4 h incubation, respectively), and the observed cellular uptake (6–18%) was due essentially to the surface-bound fraction (5–17%, Figure S19). Lipophilic molecules are usually more prone to passively diffuse through the cell membrane than the hydrophilic congeners. The lipophilicity of the tested ^99m^Tc complexes, measured as Log P_o/w_ values, varies in the order **^99m^Tc-TPP** > **^99m^Tc-TPP-BBN** > **^99m^Tc-BBN**. Therefore, the occurrence of passive transport in the cellular uptake of these radiocomplexes is unlikely as the more lipophilic **^99m^Tc-TPP** showed a negligible internalization when compared with the BBN-containing molecules. This is also corroborated by the lower internalization that was found for **^99m^Tc-TPP-BBN** (Log P_o/w_ = 0.45) when compared with **^99m^Tc-BBN** (Log Po/w = −0.75).

**Figure 5:**
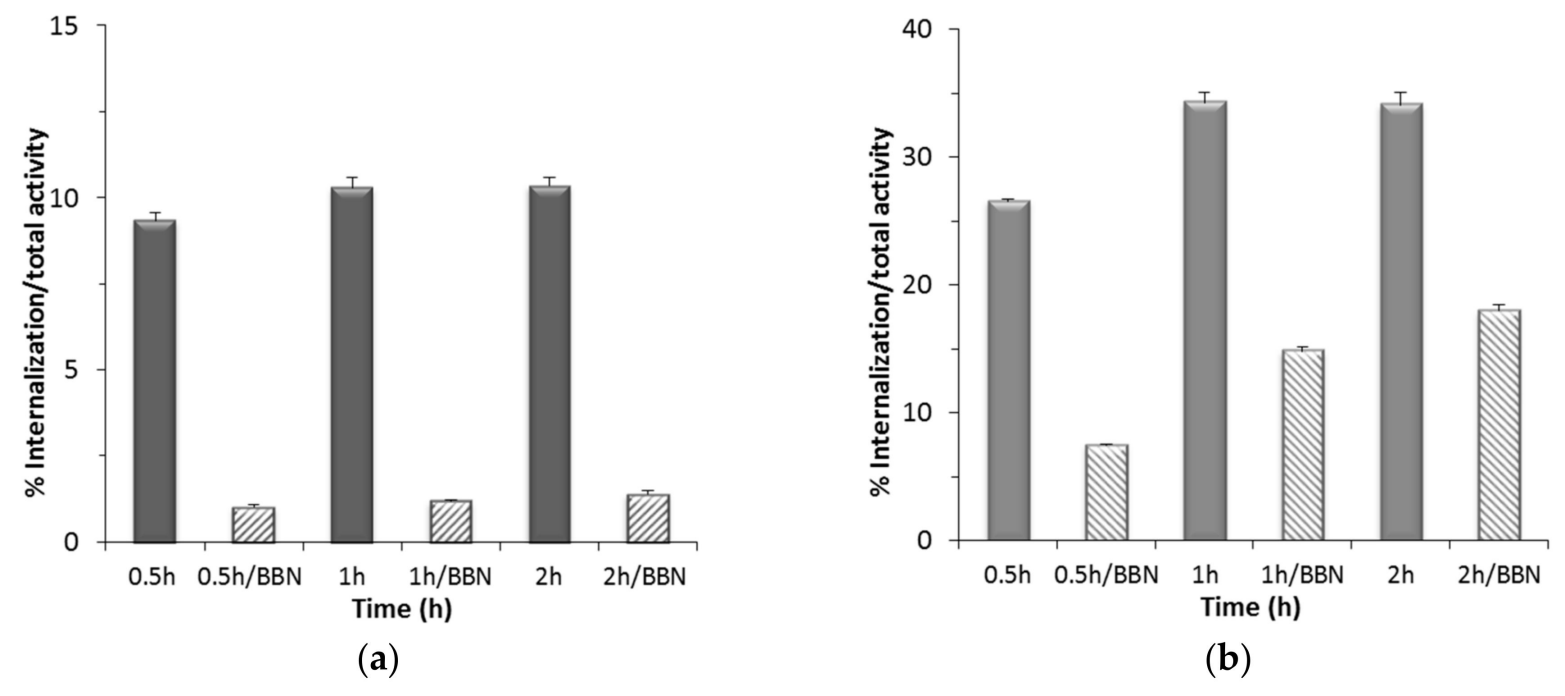
GRP receptor-blocking study: inhibition of internalization of ^99m^Tc-TPP-BBN (**a**) and ^99m^Tc-BBN (**b**) in PC3 cells, at 37 °C, by coincubation with [Tyr^4^]-BBN (0.25 µg/0.5 mL/well). Data was expressed as a percentage of the total (applied) activity. Results were calculated from independent biological replicates (*n* = 4), and are given as the average ± SEM.

To check whether the cellular internalization of **^99m^Tc-BBN** and **^99m^Tc-TPP-BBN** was mediated by the GRP receptor, these radioconjugates were incubated with PC3 cells in the presence of cold [Tyr^4^]-BBN to block the GRPr. The cellular internalization results, in the presence and the absence of [Tyr^4^]-BBN, are shown in Figure 5. The effect of the receptor blockade in the uptake, surface-bound fraction, and internalization is also represented in Figure S20, as the percentage of inhibition.

The co-incubation with an excess of [Tyr^4^]-BBN, a potent GRPr agonist, extensively inhibited the cellular internalization of **^99m^Tc-TPP-BBN** by 87–89%, as can be seen in Figure 5a and Figure S20a. The excess of [Tyr^4^]-BBN also reduced in 26 to 35% the surface-bound level of **^99m^Tc-TPP-BBN**, being the strongest inhibition observed for shortest incubation time (0.5 h). In the case of **^99m^Tc-BBN**, the internalization and binding to the cell membrane were also significantly blocked (by 71% and 11%, respectively), when incubated for 0.5 h in the presence of [Tyr^4^]-BBN (Figure 5b and Figure S20b). However, this blockade was not so extensive and durable as the one observed for **^99m^Tc-TPP-BBN**. These differences, as well as the highest internalization rate exhibited by **^99m^Tc-BBN**, seem to indicate that **^99m^Tc-TPP-BBN** and **^99m^Tc-BBN** might have different affinity towards the GRPr. Nevertheless, altogether, the obtained cellular results indicate that both radiocomplexes are mostly taken up by prostate cancer cells through a GRPr-mediated mechanism.

#### 2.3.3. Mitochondria Uptake

**Figure 6:**
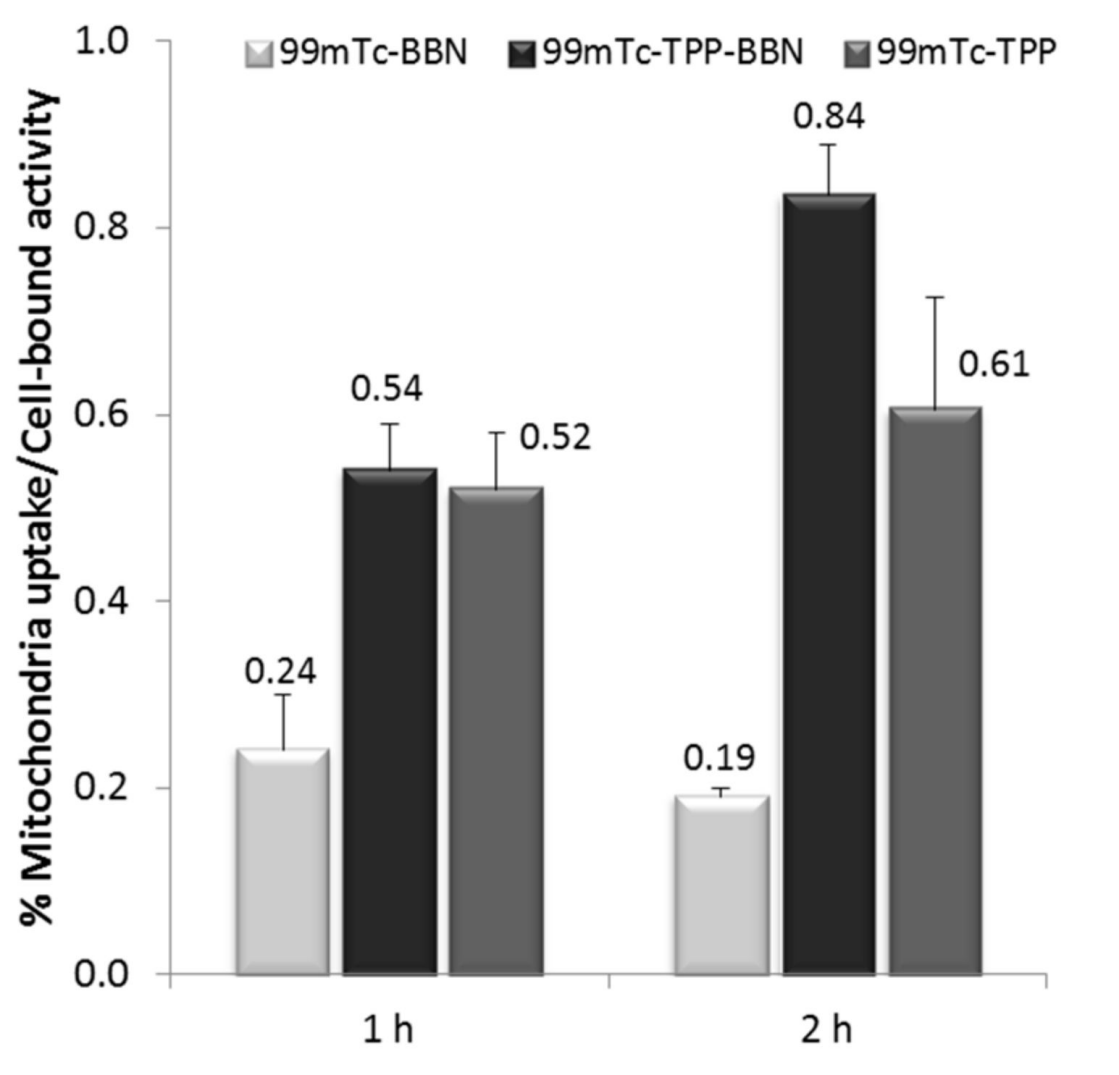
Relative uptake of **^99m^Tc-TPP-BBN**, **^99m^Tc-TPP** and **^99m^Tc-BBN** into mitochondria compared to whole cell uptake, after 1 h and 2 h of incubation in PC3 cells. Results were calculated from independent biological replicates (*n* = 6), and are given as the average ± SEM.

In order to verify if the dual-targeted complex **^99m^Tc-TPP-BBN** was able to reach the mitochondria, its subcellular localization and distribution was evaluated in PC3 cells, after 1 h and 2 h of incubation. For comparison, parallel studies were done with the related single-targeted radiocomplexes **^99m^Tc-TPP** and^**99m**^**Tc-BBN** with or without the mitochondrion-tropic moiety. After removal of unbound radiocomplex by PBS washing, the cells were lysed and the mitochondria were extracted, as described in detail in the experimental part. Thereafter, the radioactivity in the mitochondria pellet was measured by a dose calibrator and expressed relatively (%) to the activity in the whole cell pellet. The obtained results are represented in Figure 6.

The **^99m^Tc-BBN** complex accumulates to a low extent (0.19 to 0.24%) in mitochondria when compared with the TPP-containing complexes. Despite its low degree of cellular internalization, the **^99m^Tc-TPP** accumulates 2–3 times more in mitochondria when compared with **^99m^Tc-BBN**. The highest mitochondrial uptake (0.84 ± 0.06%) was found for the dual-targeted complex, **^99m^Tc-TPP-BBN**, after 2 h of incubation. The measured uptakes are relative values that were obtained in the same manner and, therefore, reflect the mitotropic properties of each complex. However, it is worthwhile to mention that in these type of assays, the mitochondria structure can be damaged and its content released into other subcellular components like the cytosol, nuclei, and cellular fragments, as we and other authors have confirmed [17,39]. For instance, the lipophilic cation ^99m^Tc-TMEOP had an accumulation of 2.56% in the mitochondria fraction isolated from rat myocardium that increased by more than 73% upon appropriate correction due to the presence of mitochondria in other cellular fractions. In our hands and using the same assay, the cardiac imaging agent ^99m^Tc-MIBI with well-recognized mitochondrion-tropic properties showed a measured 2.38% mitochondrial uptake that augmented to 68%, after the same type of correction. These corrections involve the assessment of the influence of the mitochondrial uncoupler CCCP in the activity retained in each isolated cellular fraction in combination with the measurement of the enzymatic activity of malate dehydrogenase in each of these fractions, which can be correlated with the presence of mitochondrial enzymatic content as a result of mitochondrial damage during the subcellular fractionation assays. Although we did not perform such correction in the present study, we are confident that the obtained results show the correct trend on the mitochondrial affinity of the tested radiocomplexes, as the itochondrial uptake of each radiocomplex should be equally affected by the eventual correction. Moreover, the mitochondrial uptake of 0.84% measured for the dual-targeted **^99m^Tc-TPP-BBN** indicates that this complex has a significant accumulation in this organelle, taking into consideration the values that we have previously found for ^99m^Tc-TMEOP and ^99m^Tc-MIBI.

#### 2.3.4. Preliminary Radiobiological Studies: Clonogenic Assay

One of the goals of this study was to demonstrate that an augmented accumulation of ^99m^Tc complexes in the mitochondria of tumor cells should enhance their radiotoxic effects, due to the high radiosensitivity of this organelle and the emission of short-range and high LET Auger electrons by ^99m^Tc. In an attempt to verify this concept, we evaluated the biological effects on PC3 cells upon incubation with increasing activities of the complexes **^99m^Tc-BBN and ^99m^Tc-TPP-BBN**, which showed a high and specific internalization in this cell line but quite different mitochondrial uptake. For this purpose, we used the clonogenic cell survival assay that has been applied over decades to assess the radiosensitivity of cancer cells when exposed to high LET radiation, giving important hints to tumor responses to radiation therapy [40].

**Figure 7:**
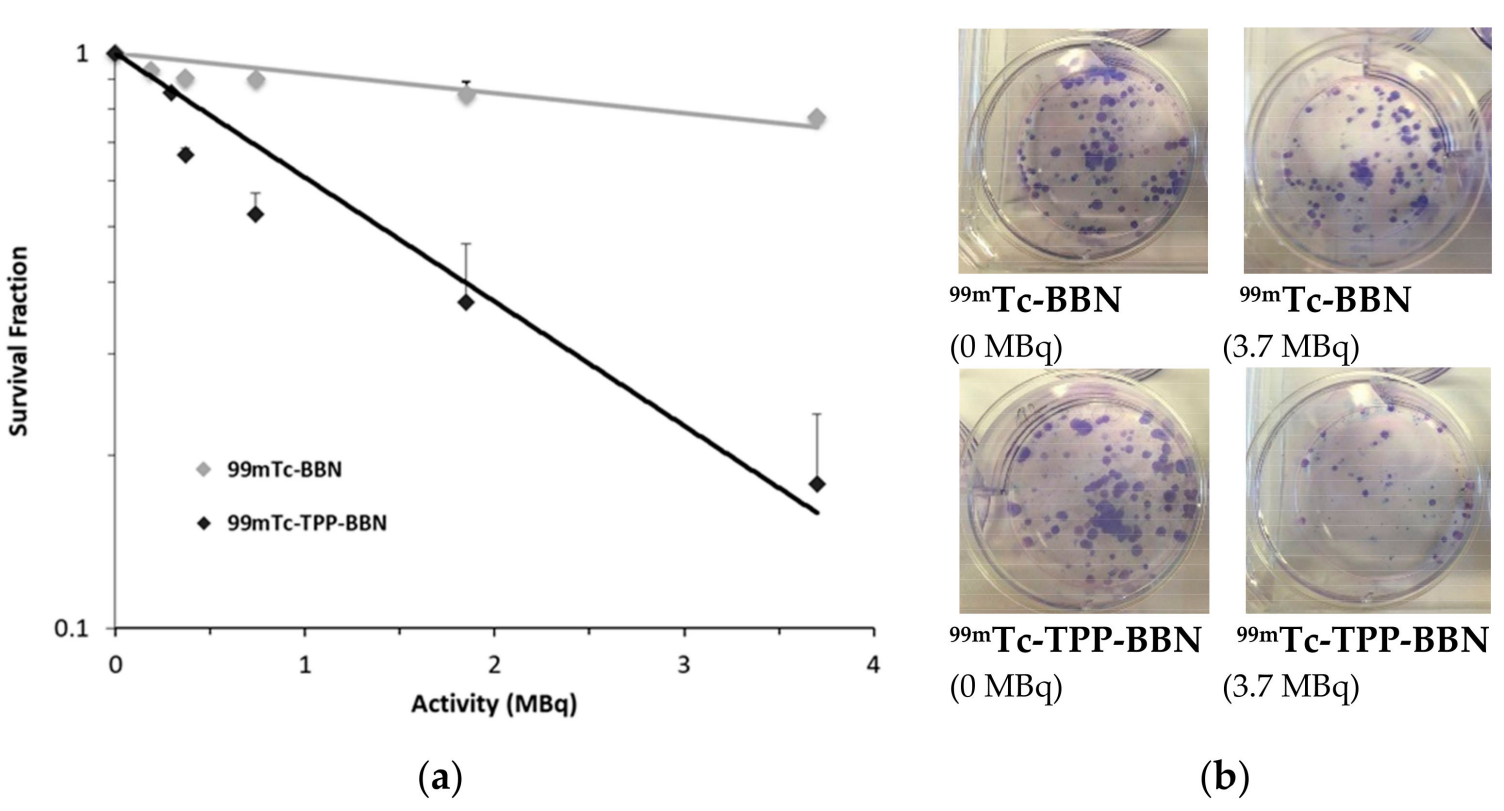
Clonogenic assay of HPLC purified **^99m^Tc-TPP-BBN** and **^99m^Tc-BBN** (0.37–3.7 MBq) in PC3 cells. (**a**) Quantification of the assay (survival fraction). The data correspond to means ± standard deviations of 3 replicates. (**b**) Representative images of single-cell clone proliferation, stained with crystal violet.

Hence, PC3 cells were incubated with increasing amounts of the radiocomplexes and the surviving fractions as a function of activity were determined using the clonogenic assay. After treatment, cells were plated in different dilutions and left to form colonies for 10 days. As can be seen in Figure 7, an activity-dependent clonogenic survival curve was clearly found for the dual targeted **^99m^Tc-TPP-BBN**. By contrast, the survival rate of PC3 cells exposed to **^99m^Tc-BBN** was only slightly affected by the applied activity up to a maximum of 3.7 MBq. Moreover, for all the tested activities, the highest cytotoxicity was observed for the dual-targeted **^99m^Tc-TPP-BBN** complex.

The highest ability of **^99m^Tc-TPP-BBN** to compromise PC3 cell survival is evident by the LD_50_, LD_20_, and LD_10_ values that were calculated from the clonogenic assay data and expressed as the activities required for 50%, 20%, and 10% reduction of the cell survival (Table 1). The LD values determined for **^9m^Tc-TPP-BBN** were about six times lower than the values found for **^99m^Tc-BBN**, thereby showing the greater effectiveness of the **^99m^Tc-TPP-BBN** to cause radiation-induced damage in PC3 cells.

## 3. Discussion

The examples of dual-targeted Auger-electron emitting radioconjugates, directed towards extracellular cancer targets and intracellular organelles, remain scarce. As mentioned in the introduction, we have previously reported M-AO-BBN (M = Re, ^99m^Tc) complexes, carrying also a triglycine cleavable linker, which exhibited a prominent cell-specific nuclear internalization [5]. Roger Alberto and co-workers described similar Re and ^99m^Tc complexes combining also a BBN derivative with the DNA intercalator acridine orange (AO). Unlike our M-AO-BBN (M = Re, ^99m^Tc) complexes, the Alberto’s dual-targeted compounds were unable to significantly accumulate in the nucleus of prostate cancer cells although showing a high level of internalization into the cytoplasm by receptor-mediated endocytosis [41,42].

A series of ^111^In-labeled somatostatin derivatives carrying a nuclear localization sequence (NLS), to transport ^111^In to the cell nucleus, were submitted to an in vitro evaluation in a pancreatic tumoral cell line, a much higher accumulation of the radionuclide in the cell nuclei being observed in some cases when compared with congener radioconjugates without NLS [43]. More recently, Chastel et al. evaluated multifunctional neuropeptide-Y (NPY) conjugates containing the glycine-leucine-phenylalanine-glycine (GLFG) sequence as a cathepsin B cleavable linker. The presence of the linker was expected to promote the release of a DOTA-NLS unit labeled with ^111^In for enhanced internalization in the nucleus of tumor cells. Encouraging results were reported with a time-dependent and high nuclear uptake of ^111^In in breast cancer MCF-7 cells, which led the authors to consider that this class of multifunctional radioconjugates represent a promising concept for enhanced TRT with Auger electron-emitting radiolanthanides [43]. However, so far, the study of radiobiological effects by these multifunctional complexes was not reported.

Similarly to the approach explored in this work, Mindt et al. described a ^99m^Tc-tricarbonyl-labeled radioconjugate comprising a modified BBN amino acid sequence ([Nle^14^]BBN[7-14]) for extracellular targeting of GRPr and a TPP moiety for accumulation in mitochondria [44]. Receptor-specific cell internalization was confirmed for this dual-targeted radioconjugate but the intracellular targeting of mitochondria could not be confirmed. This is probably the result of the hindered passage of the TPP moiety through the membrane of the organelle due to the attachment to the peptide, a problem also observed for cell penetrating peptide/TPP conjugates [45].

In this contribution, we described novel pyrazolyl-diamine Re(I)/^99m^Tc(I) tricarbonyl complexes functionalized with a mitotropic TPP moiety, to promote accumulation of the metal/radiometal in the mitochondria, and with a bombesin analogue (G3BBN[7-14]) for specific recognition of PCa tumor cells overexpressing the GRPr. The designed dual-targeted complexes, **M-TPP-BBN** (M = Re, ^99m^Tc), also contain a cathepsin B cleavable linker (Gly-Gly-Gly) between the pyrazolyl-diamine chelator framework and the BBN peptide. In this way, we expected to promote the intracellular release of smaller-sized radiocomplexes carrying the TPP and a more efficient targeting of the mitochondria with enhanced radiobiological effects by ^99m^Tc. These multifunctional complexes with distinct biological targeting abilities were synthesized efficiently, using convergent and straightforward synthetic approaches and profiting from the versatility of pyrazolyl-diamine chelators [32,33,34]. For comparative purposes, the related single-targeted **M-TPP-BBN** and **M-TPP** (M = Re, ^99m^Tc) complexes were also synthesized and evaluated.

Both the dual-targeted and single-targeted ^99m^Tc(I) complexes described in this work, **^99m^Tc-TPP-BBN**, **^99m^Tc-BBN** and **^99m^Tc-TPP**, were obtained in high yield and high radiochemical purity (RCP > 95%) after purification by HPLC. Their chemical identities were ascertained by HPLC comparison with authentic samples of the Re congeners that were characterized by common analytical techniques. The **^99m^Tc-TPP** complex exhibited excellent in vitro stability (RCP > 95% after 24 h of incubation in PBS, at 37 °C) while the **^99m^Tc-TPP-BBN** and **^99m^Tc-TPP-BBN** radioconjugates presented a teeny tendency to undergo oxidation at the methionine residue. Nevertheless, more than 90% of the radiocomplexes remain intact in PBS solution even after 24 h at 37 °C, which allowed their further biological evaluation.

The **^99m^Tc-TPP-BBN** and **^99m^Tc-BBN** complexes showed high and receptor-mediated cellular uptake and internalization on GRPr-positive PC3 cells. This result showed that the inclusion of the TPP group did not compromise the ability of the dual-targeted complex to recognize the GRPr. In contrast, **^99m^Tc-TPP** presented low cellular uptake and negligible internalization in the same cell line. For **^99m^Tc-TPP**, a more important membrane-bound fraction was found that certainly reflects its dicationic character, moderate lipophilicity and absence of targeting peptide. Unlike **^99m^Tc-BBN**, both complexes carrying the TPP group were able to target the mitochondria, once inside the cell. Interestingly, the dual-targeted **^99m^Tc-TPP-BBN** is the complex that showed the highest fraction of intracellular activity associated with the mitochondria. This result confirmed that **^99m^Tc-TPP-BBN** retained mitochondrion-tropic properties despite the presence of the BBN peptide, and contrasts with the results reported earlier by Mindt et al. for a related TPP-containing ^99m^Tc-radioconjugate [44]. In the latter case, the presence of the targeting peptide [Nle^14^]BBN[7-14] hampered the mitochondria accumulation, as mentioned above. Most probably, the presence of the Gly-Gly-Gly cleavable linker in **^99m^Tc-TPP-BBN** leads to the formation of smaller-sized ^99m^Tc-complexes containing the TPP moiety with enhanced ability to diffuse into the mitochondria. This possibility is sustained by the results of enzymatic assays conducted with the model **Re-BBN** complex that demonstrated the ability of cathepsin B to recognize the Gly-Gly-Gly linker with the formation of smaller-sized Re complexes, bearing two or three glycine residues, as a result of the enzymatic action.

We have considered that Auger-electron emitting radioconjugates with enhanced mitochondrial uptake in specific cancer cells could represent innovative tools for Auger electron therapy of cancer. Due to the high-energy deposition and ultrashort trajectories of Auger electrons, these radioconjugates should lead to potentially lethal damage in malignant cells while sparing the surrounding healthy tissues/cells. In this context, we decided to evaluate the radiobiological effects induced by **^99m^Tc-TPP-BBN** and **^99m^Tc-BBN** in human prostate cancer PC3 cells. As discussed above, both complexes showed high and relatively comparable cellular internalization in this cell line but quite different mitochondrial uptake. To assess the radiobiological effects, we used the clonogenic assay. The results showed that the dual-targeted complex **^99m^Tc-TPP-BBN** has a remarkably enhanced ability to reduce the cell survival for all tested activities, which certainly reflects its highest mitochondria accumulation when compared with **^99m^Tc-BBN**.

## 4. Materials and Methods

Unless otherwise stated, all chemicals and solvents were of reagent grade and used without further purification. Compound 3, *tert*-Butyl *N*-(2-{[(*tert*-butoxy)carbonyl](2-{4-[2-(2,5-dioxopyrrolidin-1-yl)-2-oxoethyl]-3,5-dimethylpyrazol-1-yl}ethyl)amino}-ethyl)carbamate, was prepared as described in the literature [35]. Compound 6, ethyl 4-((2-((*tert*-butoxycarbonyl)amino)ethyl)(2-(4-(2-((2,5-dioxopyrrolidin-1-yl)oxy)-2-oxoethyl)-3,5-dimethyl-1*H*-pyrazol-1-yl)ethyl)amino)butanoate, was prepared according to published methods.[5] The starting material *fac*-[Re(H_2_O)_3_(CO)_3_]Br was synthesized according to the literature [46]. Complex Re-Pz-COOH was synthesized as described elsewhere [47]. The G3BBN[7-14] derivatives Pz-BBN and Re-BBN were prepared as previously described [5]. Na[^99m^TcO_4_] was eluted from a commercial ^99^Mo/^99m^Tc generator, using 0.9% saline solution. The radioactive precursor *fac*-[^99m^Tc(CO)_3_(H_2_O)_3_]^+^ was prepared as described in the literature [5,6].

^1^H-, ^13^C- and ^31^P-NMR spectra were recorded on a Bruker Avance III 400 MHz or 300 MHz spectrometers. The chemical shifts (δ) are given in ppm and were referenced to the residual solvent resonances relative to tetramethylsilane (SiMe_4_) and the ^31^P chemical shifts were referenced with external 85% H_3_PO_4_ solution. Coupling constants (*J*) are given in Hz.

Mass spectra were acquired in an electrospray ionization/quadrupole ion trap (ESI/QITMS) Bruker HCT mass spectrometer (Bruker, Billerica, MA, USA). Samples were injected in mixtures of water:acetonitrile or water:methanol and injected at a flow rate of 150 µL·h^−1^.

Column chromatography was performed with silica gel 60 (Merck). HPLC (Perkin Elmer, Waltham, MA, USA) analysis of the Re and ^99m^Tc complexes was performed on a Perkin–Elmer LC pump 200 coupled to a LC 290 tunable UV-Vis detector and to a Berthold LB-509 radiometric detector, using an analytic Macherey-Nagel C18 reversed-phase column (Nucleosil 100–10, 250 × 4 mm) with a flow rate of 1 mL/min. HPLC solvents consisted of 0.1% CF_3_COOH in H_2_O (eluent A) and 0.1% CF_3_COOH in acetonitrile (eluent B) and two different gradients were used. In method 1, the following gradient was used: t = 0–3 min, 0% eluent B; 3–3.1 min, 0–25% eluent B; 3.1–9 min, 25% eluent B; 9–9.1 min, 25–34% eluent B; 9.1–20 min, 34–100% eluent B; 20–25 min, 100% eluent B; 25–26 min, 100–0% eluent B; 26–30 min, 0% eluent B. In method 2 the following gradient was used: t = 0–3 min, 0% eluent B; 3–3.1 min, 0–25% eluent B; 3.1–9 min, 25% eluent B; 9–9.1 min, 25–34% eluent B; 9.1–25.1 min, 34–54% eluent B; 25.1–26 min, 0% eluent B; 26–30 min, 0% eluent B.

### 4.1. Synthesis of Triphenylphosphonium Derivatives

#### 4.1.1. (3-Phtalimidylpropyl) triphenylphosphonium (**1**)

A mixture containing bromopropyl phtalamide (6.33 g, 0.024 mol) and triphenylphosphine (6.11 g, 0.024 mol) in acetonitrile (70 mL) was refluxed for 24 h. The progress of reaction was monitored by TLC (DCM/EtOH; 90/10). After cooling, the solvent was evaporated under reduced pressure and the crude product was purified by column chromatography on silica gel using as eluents DCM/EtOH (100% DCM followed by 80/20 (DCM/EtOH)). The solvent was evaporated to afford a white solid (9.9 g, 0.022 mol, 91.8%). ^1^H-NMR (300 MHz, CDCl_3_) δ_H_ 7.86–7.64 (m, 15H, CH, Ar(PPh_3_)+ 4H, CH, Ar(Pth)), 4.07–4.00 (m, 2H, C*H*_2_), 3.95 (t, 2H, CH_2_), 2.16–2.06 (m, 2H, CH_2_). ^31^P-NMR (MHz, CDCl3) δ_P_ 24.76. ESI(+)-MS *m*/*z* calcd for [C_29_H_25_NO_2_P]^+^: 450.17, found: 450.3 [M]^+^.

#### 4.1.2. (3-Aminopropyl)triphenylphosphonium (**2**)

To a solution of 1 (3.13 g, 6.9 mol) in EtOH (35 mL) was added hydrazine hydrate 50–60% (2.7 g). The reactional mixture was refluxed for 18 h. After cooling, 3M HCl was added to the mixture (pH 3–4) and the mixture was stirred for 15 min leading to the formation of a white precipitate. The solids were separated by filtration and the pH of the filtrate was adjusted to 9–10 by adding an appropriate volume of 10 M NaOH solution. After the basification, an extraction was performed using water and CHCl_3_. After extracting the product from the aqueous phase 3 times with CHCl_3_, the combined organic phases were dried with MgSO4, filtered and the filtrate was evaporated to dryness under reduced pressure to afford 2 as a white solid (1.85 g, 84%).

^1^H-NMR (300 MHz, CD_3_OD) δ_H_ 7.91–7.56 (m, 15H, CH, Ar-PPh_3_), 3.53–3.46 (m, 2H, C*H*_2_), 2.95 (t, 2H, CH_2_), 1.93–1.83 (m, 2H, CH_2_). ^13^C-NMR (100 MHz, CD_3_OD) δc 136.42 (d, *J*_P-C_ = 3.0 Hz, PPh_3_), 134.85 (d, *J*_P-C_ = 10.0 Hz, PPh_3_), 131.62 (d, *C* = 12.6 Hz, PPh_3_), 119.67 (d, *J*_P-C_ = 86.7 Hz, PPh_3_), 42.12 (d, *J*_P-C_ = 19.4 Hz, CH_2_), 25.10 (s, CH_2_), 20.60 (d, *J*_P-C_ = 53.5 Hz, CH_2_). ^31^P-NMR (MHz, CDCl3) δ_P_ 23.60. ESI(+)-MS *m*/*z* calcd for [C_21_H_23_NP]^+^: 320.16, found: 320.2 [M]^+^.

#### 4.1.3. *tert*-Butyl(2-((*tert*-butoxycarbonyl)amino)ethyl)(2-(3,5-dimethyl-4-(2-oxo-2-((3-(triphenylphosphoranyl)propyl)amino)ethyl)-1*H*-pyrazol-1-yl)ethyl)carbamate (**4**)

To a solution of compound 3 (0.194 g, 0.36 mmol) in CH_2_Cl_2_ (10 mL) was added compound 2 (0.128 g, 0.4 mmol) in CH_2_Cl_2_ (10 mL) and DIPEA (320 μL). The reaction mixture was stirred overnight at room temperature and the progress was monitored by TLC (DCM/MeOH; 90/10). After the reaction was completed, the solution was washed with water (3 times). The organic phase was collected, dried with MgSO_4_, filtered, and the solvent evaporated to afford product 4 as a yellow oil (0.28 g, 100%).

^1^H-NMR (300 MHz, CDCl_3_) δ_H_ 7.80–7.62 (m, 15H, CH, Ar-PPh_3_), 4.07–4.01 (m, 2H, CH_2_), 3.72 (t, 2H, CH_2_), 3.47–3.44 (m, 4H, C*H*_2_), 3.39 (s, 2H, CH2), 3.13–3.07 (m, 4H, CH_2_), 2.57–2.52 (m, 2H, CH_2_), 2.16 (s, 6H, CH_3_), 1.40–1.29 (s, 18H, Boc). ^31^P-NMR (MHz, CDCl3) δ_P_ 24.39. ESI(+)-MS *m*/*z* calcd for [C_42_H_57_N_5_O5P]^+^: 742.41, found: 742.8 [M]^+^.

#### 4.1.4. 2-(1-(2-((2-Aminoethyl)amino)ethyl)-3,5-dimethyl-1*H*-pyrazol-4-yl)-*N*-(3-(triphenylphosphoranyl)propyl)acetamide (**TPP-Pz**)

To a solution of 4 (0.20 g, 0.27 mmol) in CH_2_Cl_2_ (1 mL) was added TFA (1 mL) and the mixture stirred overnight at room temperature. The solvents were evaporated in a vacuum line and the residue washed several times with ethanol, purified by Sep-Pak C18 cartridge and freeze-dried affording TPP-Pz (0.11 g; 80%).

^1^H-NMR (300 MHz, CD_3_OD) δ_H_ 7.87–7.67 (m, 15H, CH, Ar-PPh_3_), 4.30 (t, 2H, CH_2_), 3.68 (m, 2H, CH_2_), 3.50 (t, 2H, C*H*_2_), (m, 4H, CH_2_), 3.39 (s, 2H, CH2), 3.18 (q, 2H, CH_2_), 2.14 (s, 3H, CH_3_), 2.03 (s, 3H, CH_3_), 1.82 (m, 2H, CH_2_).

^13^C-NMR (75 MHz, MeOD) δ_C_ 172.82 (s), 147.83 (s), 138.93 (s), 135.02 (d, *J* = 3.0), 133.42 (d, *J* = 10.0), 130.22 (d, *J* = 12.6), 118.84 (s), 117.70 (s), 110.52 (s), 54.41 (s), 44.37 (s), 43.63 (s), 42.40 (s), 39.25 (d, *J* = 19.3), 36.55 (s), 35.46 (d, *J* = 3.3), 29.92 (s), 22.34 (d, *J* = 3.8), 19.72 (s), 19.01 (s), 17.33 (s), 15.90 (s), 11.76 (s), 10.55 (s), 8.07 (s). ^31^P-NMR (MHz, CDCl3) δ_P_ 23.58. ESI(+)-MS *m*/*z* calcd for [C_32_H_41_N_5_OP]^+^: 542.30, found: 542.6 [M]^+^.

#### 4.1.5. Ethyl 4-((2-((*tert*-butoxycarbonyl)amino)ethyl)(2-(3,5-dimethyl-4-(2-oxo-2-((3-(triphenylphospho ranyl) propyl)amino)ethyl)-1*H*-pyrazol-1-yl)ethyl)amino)butanoate (**7**)

To a solution of compound 6 (0.12 g, 0.22 mmol) and compound 2 (0.079 g, 0.24 mmol) in dry dimethylformamide (DMF; 6 mL) was added *N*,*N*-diisopropylethylamine (DIPEA; 60 µL) with stirring at room temperature. After 72h, the solvent was removed under reduced pressure and the residue was purified by column chromatography, using a gradient elution from DCM/MeOH (99/1) to DCM/MeOH (90/10) to give 9 (0.115 g, 70%) as a white solid. ^1^H-NMR (400 MHz, MeOD) δ_H_ 7.98–7.87 (m, 3H, CH, Ar-PPh_3_), 7.86–7.68 (m, 12H, CH, Ar-PPh_3_), 4.10 (q, *J* = 7.1 Hz, 2H, CH_2_), 4.01 (t, *J* = 6.4 Hz, 2H, CH_2_), 3.37–3.31 (m, 4H, CH_2_), 3.25 (s, 2H, CH_2_), 3.04 (t, *J* = 6.0 Hz, 2H, CH_2_), 2.75 (t, *J* = 6.2 Hz, 2H, CH_2_), 2.53 (t, *J* = 6.4 Hz, 2H, CH_2_), 2.48 (t, *J* = 6.4 Hz, 2H, CH_2_), 2.25–2.17 (m, 5H, CH_2_+CH_3_), 2.11 (s, 3H, CH_3_), 1.86 (dd, *J* = 15.3, 7.5 Hz, 2H, CH_2_), 1.69–1.56 (m, 2H, CH_2_), 1.43 (s, 9H, 3CH_3_, boc), 1.24 (t, *J* = 7.1 Hz, 3H, CH_3_). ^13^C-NMR (100 MHz, MeOD) δ 175.41 (s), 174.27 (s), 158.31 (s), 147.59 (s), 139.58 (s), 136.38 (d, *J* = 2.9 Hz), 134.78 (d, *J* = 10.0 Hz), 131.60 (d, *J* = 12.6 Hz), 120.05 (s), 119.19 (s), 111.22 (s), 79.95 (s), 61.39 (s), 54.84 (d, *J* = 12.6 Hz), 54.50 (s), 48.27 (d, *J* = 17.9 Hz), 40.51 (d, *J* = 19.0 Hz), 39.48 (s), 32.36 (s), 31.77 (s), 28.83 (s), 23.72 (d, *J* = 7.1 Hz), 21.01 (s), 20.48 (s), 14.62 (s), 11.92 (s), 9.97 (s). ^31^P-NMR (MHz, CDCl_3_) δ_P_ 23.51. ESI(+)-MS *m*/*z* calcd for [C_43_H_59_N_5_O_5_P]^+^: 756.42, found: 756.5 [M]^+^, 378.9 [M + H]^2+^.

#### 4.1.6. 4-((2-((*tert*-Butoxycarbonyl)amino)ethyl)(2-(3,5-dimethyl-4-(2-oxo-2-((3-(triphenylphosphoranyl) propyl)amino)ethyl)-1*H*-pyrazol-1-yl)ethyl)amino)butanoic acid (**8**)

A solution of NaOH (0.016 g, 0.4 mmol) in water (2 mL) was added to a solution of compound 7 (0.1 g, 0.132 mmol) in water (8 mL) and the mixture was heated to 50–60 °C. The progress of the reaction was monitored by HPLC and was complete after 2 h. After cooling, the mixture was neutralized with 3 M HCl (80 µL) and the product was extracted from the aqueous phase 3 times with CHCl_3_. The combined organic phases were dried with MgSO_4_, filtered, and the filtrate was evaporated to dryness under reduced pressure to afford 8 (0.089 g, 92.5%) as a white solid.

^1^H NMR (300 MHz, CDCl3) δ_H_ 8.78 (s, 1H, COOH), 7.90–7.59 (m, 15H, Ar-PPh_3_), 5.98 (s, 1H, NH), 3.91 (q, 2H, CH_2_), 3.68 (t, 2H, CH_2_), 3.42(t, 2H, CH_2_), 3.33 (s, 2H, CH_2_), 3.07 (t, 2H, CH_2_), 2.70 (t, 2H, CH_2_), 2.46 (t, 2H, CH_2_), 2.32 (m, 2H, CH_2_), 2.15 (s, 3H, CH_3_), 2.11 (s, 3H, CH_3_), 1.94 (t, 2H, CH_2_), 1.80 (m, 2H, CH_2_), 1.36 (s, 9H, 3CH_3_, boc), 1.21–1.12 (m, 2H, CH_2_). ^31^P-NMR (MHz, CDCl_3_) δ_P_ 24.42. ESI(+)-MS *m*/*z* calcd for [C_41_H_55_N_5_O_5_P]^+^: 728.39, found: 728.9 [M]^+^.

#### 4.1.7. 4-((2-Aminoethyl)(2-(3,5-dimethyl-4-(2-oxo-2-((3-(triphenylphosphoranyl)propyl)amino)-ethyl)-1*H*-pyrazol-1-yl)ethyl)amino)butanoic acid (**9**)

A solution of 8 (0.039 g, 0.053 mmol) and trifluoroacetic acid (TFA; 0.77 mL) in CH_2_Cl_2_ (1.5 mL) was stirred at room temperature. The progress of reaction was monitored by HPLC. After two hours, the reaction was complete, and the solvent was removed under reduced pressure. Finally, the crude product was purified by SPE using a Sep-Pak C18 cartridge to give 9 (0.03 g, 90%) as a white solid. ^1^H-NMR (300 MHz, MeOD) δ_H_ 7.87–7.65 (m, 15H), 4.25 (t, *J* = 5.5 Hz, 2H), 3.44–3.24 (m, 9H), 3.20 (s, 2H), 2.96 (t, 2H), 2.24 (t, *J* = 6.7 Hz, 2H), 2.15 (s, 3H), 2.04 (s, 3H) 1.98 (m, 1H), 1.89–1.68 (m, 4H). ^13^C NMR (75 MHz, MeOD) δ_C_ 176.49 (s, C=O), 174.01 (s, C=O), 148.48 (s, Cpz), 140.45 (s, Cpz), 136.41 (d, *J* = 3.0 Hz,), 134.80 (d, *J* = 10.0 Hz), 131.60 (d, *J* = 12.6 Hz), 120.23 (s), 119.08 (s), 112.19 (s, C(4)Pz), 54.35 (s), 53.93 (s), 51.45 (s), 49.85 (s), 49.57 (s), 49.28 (s), 49.00 (s), 48.72 (s), 48.43 (s), 48.15 (s), 45.08 (s), 40.66 (d, *J* = 19.2 Hz), 36.64 (s), 31.31 (d, *J* = 8.4 Hz), 23.73 (d, *J* = 3.8 Hz), 21.04 (s), 20.37 (s), 11.70 (s), 9.53 (s). ^31^P NMR (MHz) δ_P_ 23.57. ESI(+)-MS *m*/*z* calcd for [C_36_H_47_N_5_O_3_P]^+^: 628.34, found: 628.8 [M]^+^.

### 4.2. Synthesis of Re(I) Complexes

General procedure: The rhenium complexes (Re-TPP and Re-TPP-COOH) were prepared by ligand exchange reaction of the precursor [Re(CO)_3_(H_2_O)_3_]Br with an equimolar amount of the selected ligand in refluxing methanol after pH adjustment (pH 6–7). The progress of the reaction was monitored by HPLC and when the reaction was complete, the solvent was removed under reduced pressure and the Re complexes were purified by HPLC (HPLC method 1) to give the desired Re(I) complex.

**Re-TPP**: Yield: 48 mg, 80%; ^1^H-NMR (300 MHz, MeOD) δ_H_ 7.89–7.62 (m, 15H, Ar), 6.91 (s br, 1H, 1NH), 5.41 (s br, 1H, 1NH), 4.50 (d, 1H, 1CH), 4.15 (t, 2H, CH_2_), 4.09 (t, 1H, 1CH), 3.86 (s br, 1H, 1NH), 3.52(m, 1H, 1CH), 3.40 (s, 2H, CH_2_), 2.84 (m, 2H, 2CH), 2.54 (m, 3H, 3CH), 2.24 (t, 2H, CH_2_), 2.35 (s, 3H, CH_3_), 2.24 (s, 3H, CH_3_), 1.98 (m, 2H, CH_2_). ^13^C-NMR (75 MHz, MeOD) δ_C_ 176.49 (s, C=O), 174.01 (s, C=O), 148.48 (s, Cpz), 140.45 (s, Cpz), 136.41 (d, *J* = 3.0 Hz,), 134.80 (d, *J* = 10.0 Hz), 131.60 (d, *J* = 12.6 Hz), 120.23 (s), 119.08 (s), 112.19 (s, C(4)Pz), 54.35 (s), 53.93 (s), 51.45 (s), 49.85 (s), 49.57 (s), 49.28 (s), 49.00 (s), 48.72 (s), 48.43 (s), 48.15 (s), 45.08 (s), 40.66 (d, *J* = 19.2 Hz), 36.64 (s), 31.31 (d, *J* = 8.4 Hz), 23.73 (d, *J* = 3.8 Hz), 21.04 (s), 20.37 (s), 11.70 (s), 9.53 (s). ^31^P-NMR (121 MHz, MeOD) δ_P_ 23.05. ESI(+)-MS *m*/*z* calcd for [C_35_H_41_N_5_O_4_PRe]^2+^: 406.62 [M]^2+^, found: 406.8 [M]^2+^. HPLC (method 1; 254 nm): Rt = 18.0 min.

**Re-TPP-COOH**: Yield: 20 mg, 60%; ^1^H NMR (300 MHz, MeOD) δ_H_ 7.93–7.75, (m, 15H, Ar), 5.53 (s br, 1H, 1NH), 4.53 (m, 1H, CH), 4.23 (m, 1H, CH), 4.06 (m, 1H, NH), 3.66 (m, 1H, CH), 3.54 (m, 2H, CH_2_), 3.48 (m, 2H, CH_2_), 3.41 (s, 2H, CH_2_), 3.38 (m, 2H, CH_2_), 2.87 (m, 2H, CH), 2.77 (m, 1H, CH), 2.69 (m, 2H, CH), 2.44 (t, 2H, CH_2_), 2.36 (s, 3H, CH3), 2.29 (s, 3H, CH3), 1.87 (m, 4H, CH_2_). ^31^P NMR (121 MHz, CD_3_CN) δ 23.99. ESI(+)-MS *m*/*z* calcd for [C_39_H_47_N_5_O_6_PRe]^2+^: 449.64 [M]^2+^, found: 449.8 [M]^2+^.

### 4.3. Synthesis of Peptide Derivatives

The G3BBN[7-14] aminoacid sequence was obtained by solid phase peptide synthesis (SPPS) methodologies using a Rink amide resin (MBHA) and employing a standard Fmoc strategy in an automated peptide synthesizer (Liberty; CEM, Matthews, NC, USA). Then, it was used to obtain the conjugates TPP-BBN and Re-TPP-BBN, as follows: 50 mg of G3BBN-containing resin (22.2 mg, 0.03 mmol) were swelled with 2 mL of DCM for 10 min. In a flask, 2 equivalents of chelator/complex were dissolved in 3 mL of DMF; 22.7 mg (0.06 mmol) of HBTU and 18 µL (0.1 mmol) of DIPEA were added, and stirred at room temperature for 10 min. The resin was filtered to remove the DCM and the chelator/complex solution was added and the reaction was stirred with N_2_ bubbling at room temperature overnight. The resin was filtered and the peptide was cleaved from the resin by using 2 × 2 mL of the TFA/Trismethylsilane/H_2_O (95%/2.5%/2.5%) mixture. The acidic solution was filtered from the resin, concentrated under N_2_ and cold diethyl ether was added, which led to the precipitation of the peptide. After centrifugation, the supernatant was removed, and the conjugated peptide purified by HPLC.

**TPP-Pz-BBN**: ESI(+)-MS *m*/*z* calcd for [C_85_H_119_N_21_O_14_SP]^+^ = 1720.87; found: *m*/*z* [M]^+^ = 1720.9; Calcd [M + H]^2+^ = 861.4; found [M + H]^2+^ = 861.7.

**Re-TPP-BBN**: ESI(+)-MS *m*/*z* calcd for [C_88_H_119_N_21_O_17_SPRe]^2+^ = 995.90; found: *m*/*z* [M]^2+^ = 996.0; [M + H]^3+^ = 665.9; HPLC (λ 254 nm): Rt = 20.8 min (method 2); Rt = 17.2 min (method 1).

### 4.4. Synthesis and In Vitro Evaluation of ^99m^Tc(I) Complexes

#### 4.4.1. Radiolabeling Procedure

To a nitrogen-purged vial containing the appropriate ligand (TPP-Pz; Pz-BBN; TPP-Pz-BBN) was added the organometallic precursor *fac*-[^99m^Tc(CO)_3_(H_2_O)_3_]^+^ in aqueous solution at pH 7. The resulting reaction mixture was then heated at 100 °C for 30 min to give the corresponding ^99m^Tc complexes (^99m^Tc-BBN; ^99m^Tc-TPP; ^99m^Tc-TPP-BBN). After cooling to room temperature, the radiochemical yield was determined by ITLC-SG using methanol/6M HCl (95/5) as eluent and also by gradient RP-HPLC analysis. All the ^99m^Tc complexes (^99m^Tc-BBN; ^99m^Tc-TPP; ^99m^Tc-TPP-BBN) were purified by HPLC to remove the excess of the respective ligand. The purified ^99m^Tc complexes were collected in 0.1 M PBS pH 7.4 containing 0.2% of BSA to avoid adsorption to the vials and the organic solvent was removed under N_2_. The chemical identity of all the ^99m^Tc complexes was established by comparison of their HPLC profiles with those of the corresponding rhenium complexes.

**^99m^Tc-BBN**: HPLC: Rt = 17.0 min (γ detection; method 1); RCP > 98% (after purification by HPLC).

**^99m^Tc-TPP**: HPLC: Rt = 18.3 min (γ detection; method 1); RCP > 98% (after purification by HPLC).

**^99m^Tc-TPP-BBN**: HPLC (γ detection): Rt = 21.4 min (method 2); Rt = 17.6 min (method 1); RCP > 95% (after purification by HPLC).

#### 4.4.2. In Vitro Stability Studies

Radiochemical stability of the ^99m^Tc complexes (^99m^Tc-TPP, ^99m^Tc-BBN and ^99m^Tc-TPP-BBN) was assessed by RP-HPLC analysis at several time points (2 h, 4 h and 24 h) following incubation in 0.1 M PBS pH 7.4 and in DMEM cell culture medium at 37 °C, respectively.

#### 4.4.3. Lipophilicity Determination

The lipophilicity of the ^99m^Tc complexes was evaluated by the “shake flask” method [48]. Briefly, 25 µL of the radiocomplexes were added to a mixture of n-octanol (1 mL) and 0.1 M PBS pH = 7.4 (1 mL), previously saturated in each other by stirring the mixture. This mixture was vortexed and centrifuged (5000 rpm, 10 min, room temperature) to allow phase separation. Aliquots of 50 µL of both octanol and PBS were counted in a gamma counter. The partition coefficient (P_o/w_) was calculated by dividing the counts in the octanol phase by those in the buffer, and the results expressed as Log P_o/w_. Log P_o/w_ = −0.75 (^99m^Tc-BBN); Log P_o/w_ = 0.52 (^99m^Tc-TPP); Log P_o/w_ = 0.45 (^99m^Tc-TPP-BBN).

### 4.5. Enzymatic Assays

A stock solution of cathepsin B from bovine spleen (Sigma Aldrich; C6286) was prepared by dissolving the lyophilized powder in 1 mL of the following buffer: 50 mM NaCl, 1 mM EDTA, 50 mM sodium acetate (pH = 5.0). This stock solution was stored at −20 °C until needed. For each assay, a portion of the enzyme stock solution (6 µL) was activated at room temperature with a solution of 30 mM DTT/15 mM EDTA (12 µL) for 15 min. To the activated cathepsin B was then added 1 mL of the buffer (pH 5.0, pre-incubated at 37 °C) and 50 µL of aqueous solution of Re-BBN (3.5 mg/mL). The reaction mixture was incubated at 37 °C, and at various time points (2 h, 4 h, 24 h, 48 h), aliquots were analysed by HPLC (method 1; λ = 254 nm). The isolated products were analysed by mass spectrometry.

### 4.6. Cell Studies

#### 4.6.1. Cell Culture

PC3 human prostate cancer cells (ECACC 90112714, England, UK) were grown in DMEM containing GlutaMax supplemented with 10% heat-inactivated fetal bovine serum and 1% penicillin/streptomycin antibiotic solution (all from Gibco, Thermo Fisher Scientific, Waltham, MA, USA), in a humidified atmosphere of 95% air and 5% CO_2_ at 37 °C (Heraeus, Hanau, Germany).

#### 4.6.2. Internalization and Cellular Uptake

Time-dependent accumulation of ^99m^Tc-TPP-BBN, ^99m^Tc-TPP and ^99m^Tc-BBN complexes in tumor cells was studied using PC3 cells. PC3 cells were seeded at a density of 0.125 million per well in 24 well-plates and allowed to attach overnight. The cells were incubated at 37 °C for a period of 5 min to 3 h with about 7.4 kBq (0.2 µCi) of the HPLC-purified radiocompound in 0.5 mL of culture medium. After each incubation time, the unbound radiocomplex was removed and the cells washed with ice-cold DMEM medium. Cell surface-bound radiocompound was removed by two steps of acid wash (50 mM glycine, HCl/100 mM NaCl, pH 2.8) at room temperature for 4 min. The pH was neutralized with cold PBS with 0.2% BSA, and subsequently the cells were lysed with 1 M NaOH for 10 min at 37 °C to determine internalized radiocompound. The activity in both cell surface-bound and internalized fractions was measured using a gamma counter (LB 2111, Berthold) and is reported as a proportion to the total applied radioactivity, together representing the cellular uptake of the radiocomplex. Assays for each time point were performed in quadruplicate and data are presented as average ± SEM of typically three independent experiments.

For assessing the specific GRPr-mediated cellular uptake and internalization of ^99m^Tc-TPP-BBN and ^99m^Tc-BBN, a similar study was performed in which radiocomplexes were incubated for 0.5 h, 1 h, and 2 h with or without the potent GRPr agonist, [Tyr^4^]-Bombesin (0.25 µg/0.5 mL/well).

#### 4.6.3. Mitochondrial Uptake

To assess the ability of ^99m^Tc-complexes to target the mitochondria, compartmental fractionation studies were performed in PC3 cells. Adherent and confluent cells (T75 culture flask) were incubated with about 7.4 MBq (100 µCi) of the HPLC purified radiocompounds in 2 mL of culture medium for 1 h and 2 h at 37 °C and 5% CO_2_. The harvested cell suspension with 10–20 × 10^6^ cells was split into 3 fractions (3 replicates, 2 mL each), centrifuged at 850× *g* for 2 min at 4 °C (Centrifuge 5804R, Eppendorf) and cells washed twice with cold PBS to remove the unbound radiocomplex and the activity of whole-cell fraction was measured (cellular uptake determination). To obtain the mitochondrial fraction, the cells were treated with the “Mitochondria Isolation Kit for Cultured cells” (Thermo Scientific, Walthman, MA, USA) according to the manufacturer’s protocol. Briefly, the pellet (one third of total pellet) was resuspended with reagent A (0.8 mL/2 × 10^7^ cells) supplemented with a cocktail of protease inhibitors (Roche, Basel, Switzerland), vortexed at medium speed for 5 s and incubated on ice for exactly 2 min. Reagent B (10 µL/2 × 10^7^ cells) was added and the samples were vortexed at maximum speed for 5 s, incubated on ice, and vortexed every minute for 5 min. Then, a reagent C (0.8 mL/2 × 10^7^ cells), supplemented with a cocktail of protease inhibitors, was added, and the cells suspension centrifuged for 10 min at 700× *g* at 4 °C (Centrifuge 5417R, Eppendorf, Hamburg, Germany). The nucleus isolation steps were monitored by trypan blue staining under a phase contrast microscope. The resulting pellet corresponds to the nuclear fraction. The supernatant, transferred to a new tube, was centrifuged for 15 min at 3000× *g* at 4 °C to remove the lysosomal and peroxisomal contaminants. The pellet, containing the isolated mitochondria, was again treated with reagent C (0.5 mL/2 × 10^7^ cells) and centrifuged for 5 min at 12,000× *g*. The activity in all fractions collected were directly counted in a dose calibrator (Berthold, LB2111, Bad Wildbad, BW, Germany).

#### 4.6.4. Clonogenic Assay

PC3 cells were seeded at a density of 50,000 cells per well in a 24 well-plate and allowed to attach overnight. Cells were incubated with several activities (0, 0.185, 0.37, 0.74, 1.85, and 3.7 MBq) of HPLC purified radioconjugates in 0.5 mL of culture medium, for 24 h at 37 °C. Immediately, after incubation, cells were seeded out in appropriate dilution to form colonies, in 2 weeks, with at least 50 cells. Colonies were fixed with methanol: glacial acetic acid (3:1) and stained with Giemsa (4%).

The Plating Efficiency (PE), ratio of the number of colonies to the number of cells seeded, and the Survival Fraction (SF), number of colonies that arise after treatment of cells, expressed in terms of PE, were obtained following the methodology described in literature [49], where:

(1) $$\mathrm{PE}=\frac{\mathrm{number}\;\mathrm{of}\;\mathrm{colonies}\;\mathrm{formed}\;}{\mathrm{number}\;\mathrm{of}\;\mathrm{cells}\;\mathrm{seeded}}\times 100\%$$

(2) $$\mathrm{SF}=\frac{\mathrm{number}\;\mathrm{of}\;\mathrm{colonies}\;\mathrm{formed}\;\mathrm{after}\;\mathrm{treatment}}{\mathrm{number}\;\mathrm{of}\;\mathrm{cells}\;\mathrm{seeded}\;\times \;\mathrm{PE}}$$

## 5. Conclusions

The dual-targeted complex **^99m^Tc-TPP-BBN** is efficiently internalized by human prostate cancer PC3 cells through a specific GRPr-mediated mechanism of uptake. Moreover, it provided an enhanced accumulation of ^99m^Tc in the mitochondria of the target tumor cells. This is most probably due to its intracellular cleavage by cathepsin B into a small-sized radiocomplex that carries the TPP moiety but not the BBN peptide. In addition, **^99m^Tc-TPP-BBN** showed an enhanced ability to reduce the survival of PC3 cells, in a dose-dependent manner.

Altogether, these results pinpoint that the proposed dual-targeted approach has the potential to enhance the outcome of anti-cancer Auger therapies. However, it is important to pursue additional and more detailed radiobiological studies to confirm local radiobiological effects on target mitochondria, namely mtDNA damage and alterations in the mitochondrial transmembrane potential or in superoxide levels. Such approach is very versatile and can be further applied to other radiometals that are more efficient Auger electron emitters than ^99m^Tc. Thus, it might open new opportunities in the design and development of radiopharmaceuticals for Auger electron therapy of cancer, by exploring tailor-made chelators fitted to each radiometal, different tumor-specific molecules and diverse mitochodrion-tropic molecules and/or cleavable linkers for a more efficient tumor retention and accumulation in the target mitochondria.

## Figures and Tables

**Table 1:** Lethal doses (LD_50_, LD_20_ and LD_10_) in PC3 cells for the **^99m^Tc-TPP-BBN** and **^99m^Tc-BBN** complexes.

Radiocomplex	LD_50_	LD_20_	LD_10_
	(MBq)	(MBq)	(MBq)
**^99m^Tc-TPP-BBN**	1.39	0.45	0.21
**^99m^Tc-BBN**	>5	2.79	1.32

## Data Availability

The data presented in this study are available in article and supplementary material.

## Supplementary Materials

The following are available online, Scheme S1: Formation of the oxidized product upon hydrolysis of compound 7, Figure S1: ^1^H-NMR spectrum of the oxidized product resulting from hydrolysis of compound 7, Figure S2: ^31^P-NMR spectrum the oxidized product resulting from hydrolysis of compound 7, Figure S3: ESI-MS spectrum of the oxidized product resulting from hydrolysis of compound 7, Figure S4: ESI-MS spectrum of the TPP-Pz in the positive ion mode, Figure S5: ESI-MS spectrum of the Re-TPP complex in the positive ion mode, Figure S6. ESI-MS spectrum of the Re-TPP-COOH in the positive ion mode, Figure S7: ESI-MS spectrum of the TPP-Pz-BBN in the positive ion mode, Figure S8: ESI-MS spectrum of the Re-TPP-BBN in the positive ion mode, Figure S9: HPLC chromatograms of inactive Re-BBN and radioactive ^99m^Tc-BBN complexes, and Pz-BBN ligand, Figure S10: RP-HPLC radiochromatograms of ^99m^Tc-BBN after incubation in PBS pH 7.4 at 37 °C for 0 h, 2 h, 4 h, and 24 h, Figure S11: RP-HPLC radiochromatograms of ^99m^Tc-BBN after incubation in DMEM at 37 °C for 0 h, 2 h, 4 h and 24 h, Figure S12: HPLC chromatograms of TPP-Pz ligand, inactive Re-TPP and radioactive ^99m^Tc-TPP complexes, Figure S13: RP-HPLC radiochromatograms of ^99m^Tc-TPP after incubation in PBS pH 7.4 at 37 °C for 2, 4, and 24 h, Figure S14: RP-HPLC radiochromatograms of ^99m^Tc-TPP after incubation in DMEM at 37 °C for 2, 4, and 24 h, Figure S15: RP-HPLC chromatograms of TPP-Pz-BBN ligand, Figure S16: RP-HPLC radiochromatograms of ^99m^Tc-TPP-BBN after incubation in PBS pH 7.4 at 37 °C for 2, 4 and 24 h, Figure S17: RP-HPLC radiochromatograms of ^99m^Tc-TPP-BBN after incubation in DMEM at 37 °C for 2, 4, and 24 h, Figure S18: RP-HPLC chromatogram of the reaction mixture obtained after incubation of Re-BBN with cathepsin B for 24 h at 37 °C, Figure S19: Time-dependent surface-bound of ^99m^Tc-TPP-BBN, ^99m^Tc-TPP and ^99m^Tc-BBN in PC3 cells at 37 °C, Figure S20: GRP receptor-blocking study for ^99m^Tc-TPP-BBN and ^99m^Tc-BBN.
