# Supplementary material for: Synthesis and Biological Evaluation of 99mTc(I) Tricarbonyl Complexes Dual-Targeted at Tumoral Mitochondria
Source: Molecules. 2021 Jan 15;26(2):441. doi: 10.3390/molecules26020441 (PMC7830118; doi:10.3390/molecules26020441)
Supplement: Supplementary file 1 [file molecules-26-00441-s001.pdf]

molecules

MDPI

Supplementary Information

I – Basic hydrolysis of compound 7 with oxidation at the TPP group

Removal of the ethyl protecting group of compound **7** using aqueous solution of NaOH under reflux overnight leads to the oxidation at the TPP group, as depicted in the scheme S1. The resulting product was separated by HPLC and characterized by ESI-MS and ^{1}H- an ^{31}P-NMR and spectra and the ESI-MS spectra in positive ion mode corroborated the structure presented bellow.

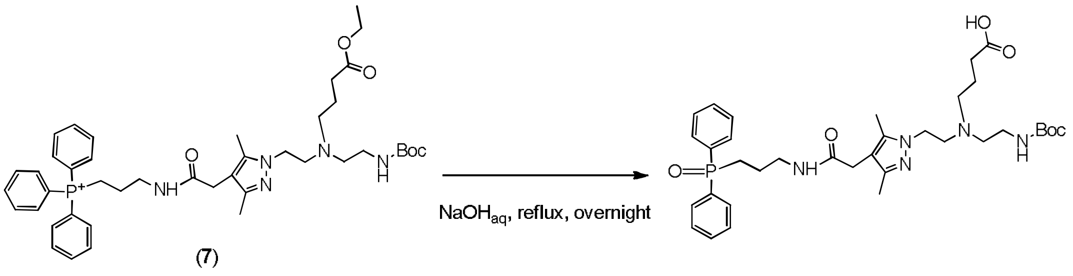

Scheme S1. Formation of the oxidized product upon hydrolysis of compound **7**, in refluxing aqueous solution of NaOH (10 eq), overnight.

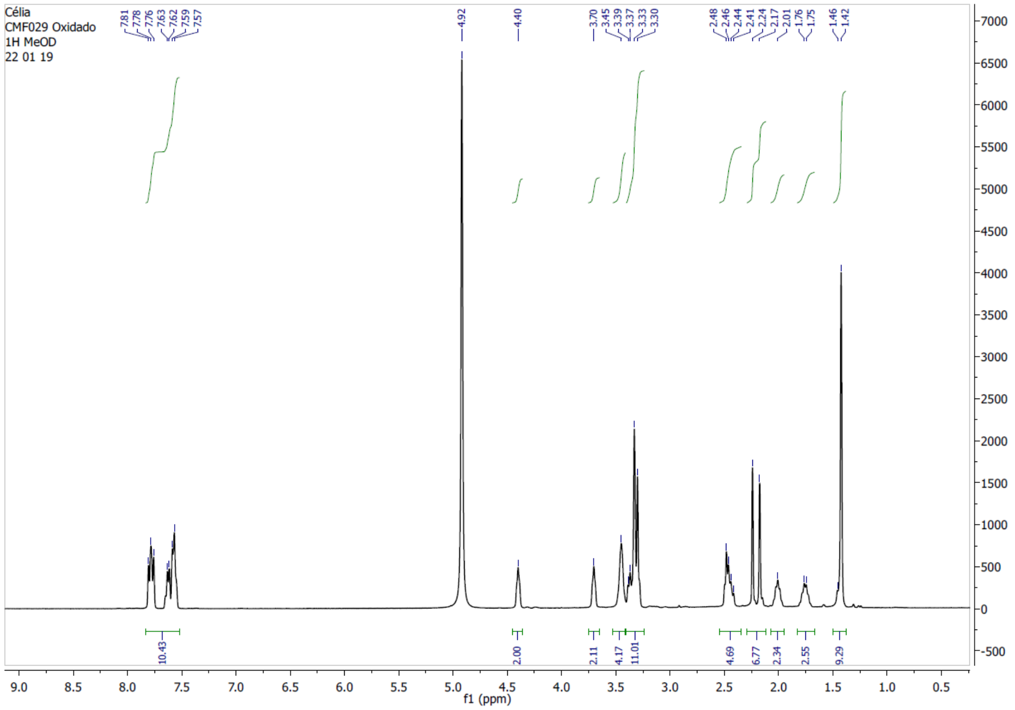

Figure S1. ^{1}H-NMR spectrum of the oxidized product resulting from hydrolysis of compound **7**.

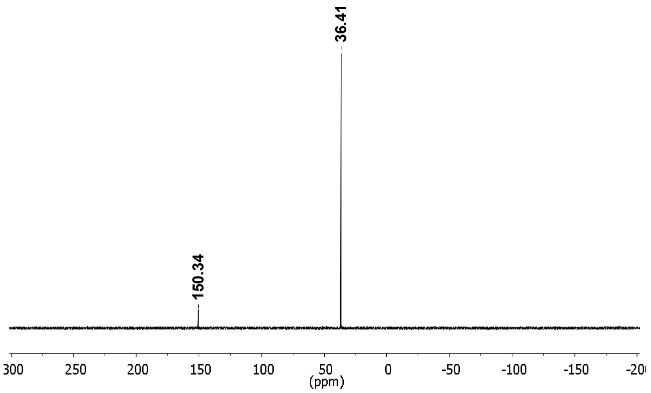

Figure S2. ^{31}P-NMR spectrum of the oxidized product resulting from hydrolysis of compound **7**.

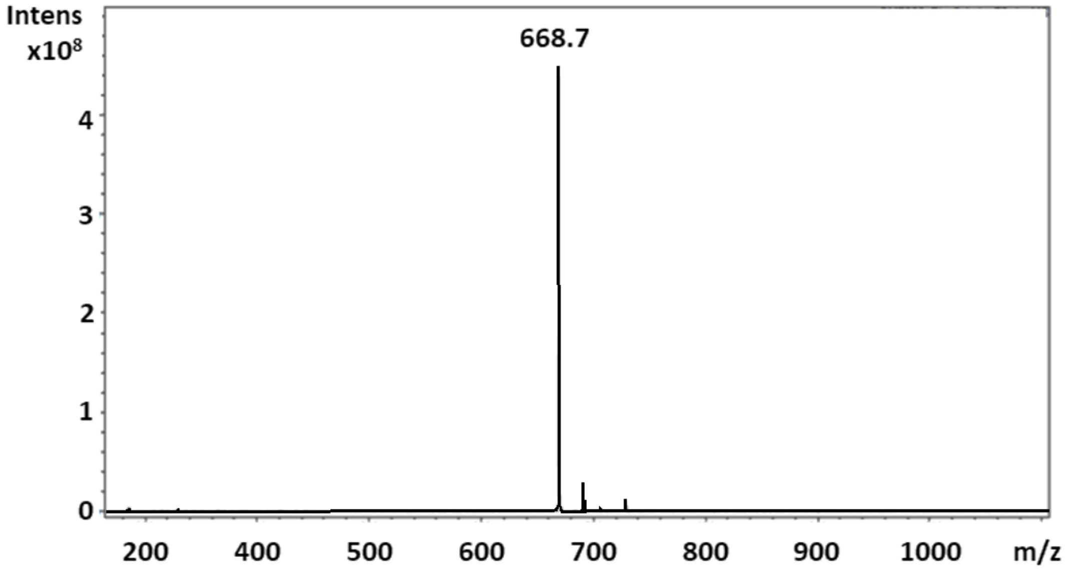

Figure S3. ESI-MS spectrum of the oxidized product resulting from hydrolysis of compound **7** (m/z calcd for $[C_{35}H_{51}N_5O_6P]^+$ = 668.36; found $[M]^+$ 668.7).

II- Chelators and Re complexes

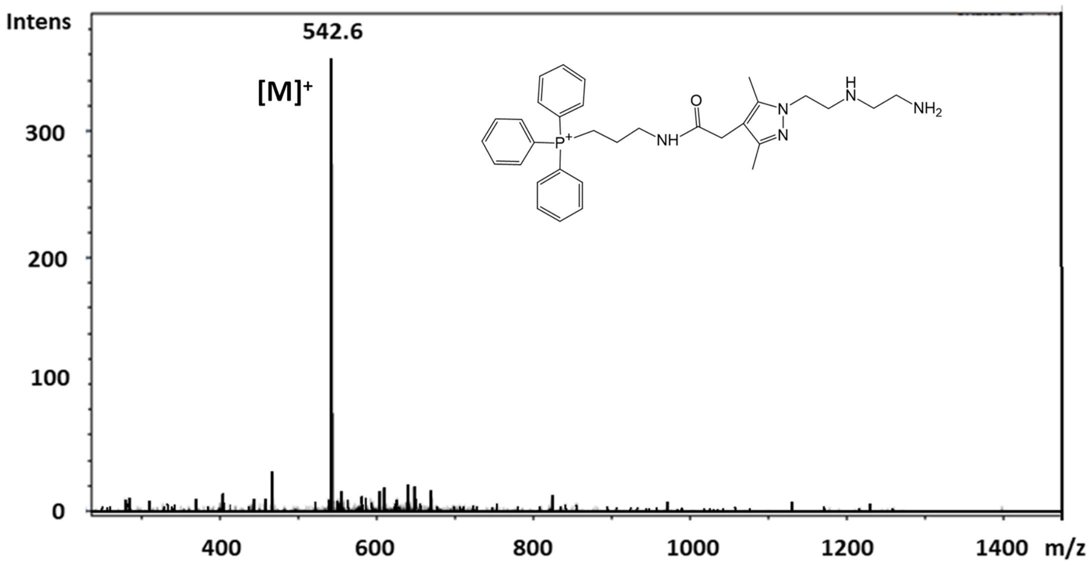

Figure S4. ESI-MS spectrum of the **TPP-Pz** in the positive ion mode (*m/z* calcd for $[C_{32}H_{41}N_5OP]^+$: 542.31 $[M]^+$, found: 542.6 $[M]^+$).

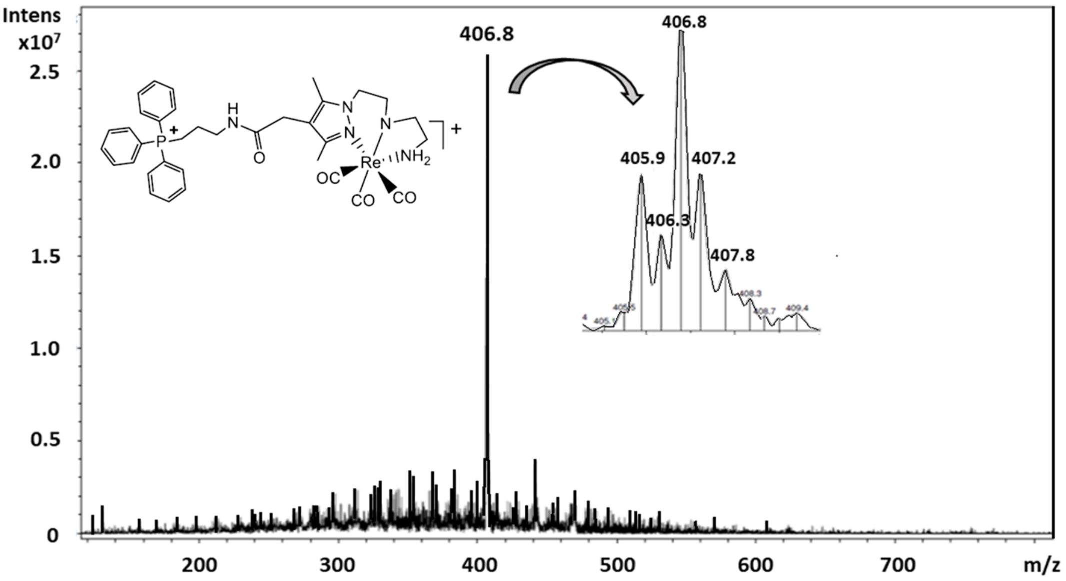

Figure S5. ESI-MS spectrum of the **Re-TPP** complex in the positive ion mode with a zoom showing the characteristic isotopic pattern (*m/z* calcd for $[C_{35}H_{41}N_5O_4PRe]^{2+}$: 406.62 $[M]^{2+}$, found: 406.8 $[M]^{2+}$).

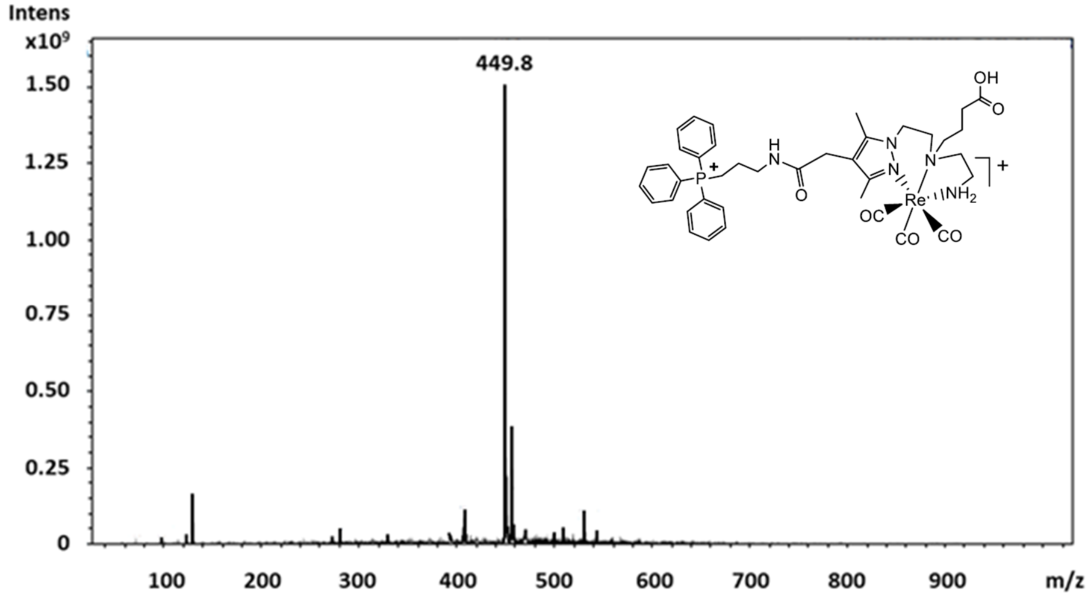

Figure S6. ESI-MS spectrum of the **Re-TPP-COOH** in the positive ion mode (*m*/*z* calcd for $[C_{39}H_{47}N_5O_6PRe]^{2+}$: 449.64 $[M]^{2+}$, found: 449.8 $[M]^{2+}$).

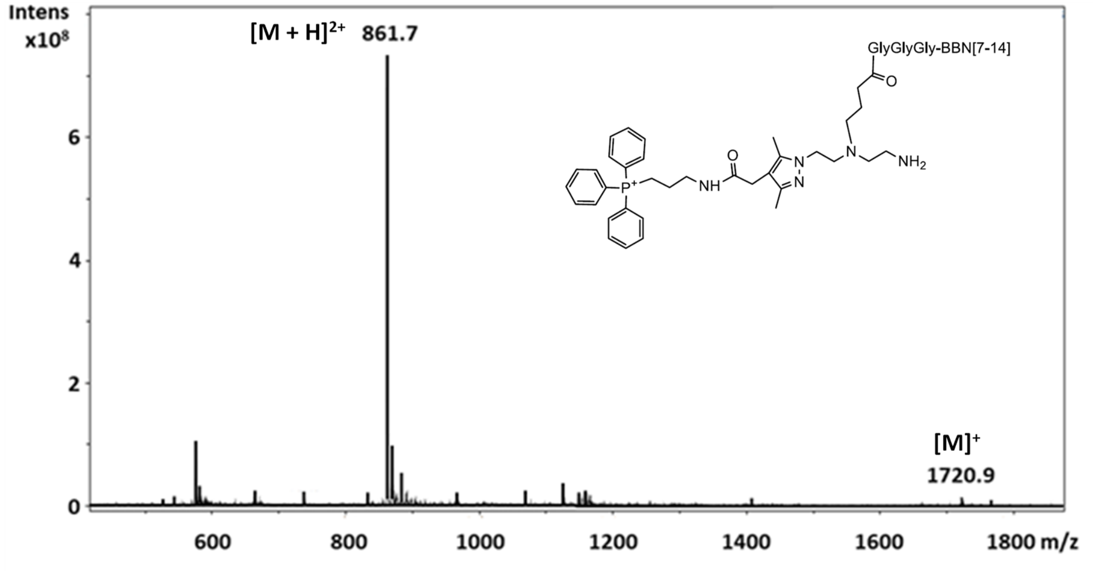

Figure S7. ESI-MS spectrum of the **TPP-Pz-BBN** in the positive ion mode (*m*/*z* calcd for $[C_{85}H_{119}N_{21}O_{14}SP]^{+}$: 1720.86 $[M]^{+}$, found: 1720.9 $[M]^{+}$, Calcd $[M+H]^{2+}$ = 861.4; found $[M+H]^{2+}$ = 861.7).

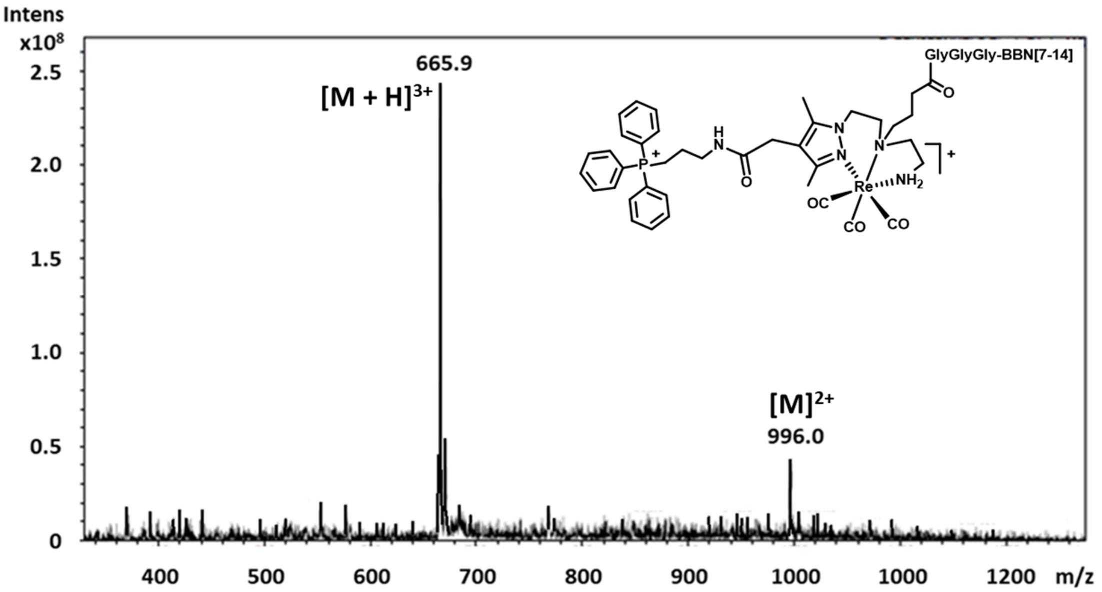

Figure S8. ESI-MS spectrum of the **Re-TPP-BBN** in the positive ion mode (*m*/*z* calcd for $[C_{88}H_{119}N_{21}O_{17}PSRe]^{2+}$: 995.90 $[M]^{2+}$, found: 996.0 $[M]^{2+}$ and 665.9 $[M+H]^{3+}$).

III- ^{99m}Tc(I) complexes: synthesis and in vitro evaluation

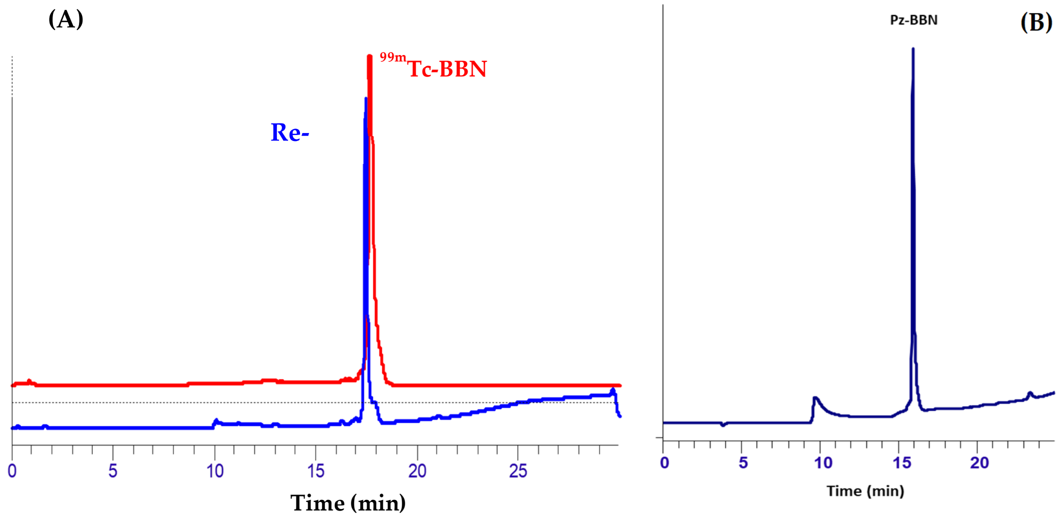

Figure S9. (A) HPLC chromatograms (method 1) of inactive **Re-BBN** (blue trace; UV detection 254 nm; Rt = 17.4 min) and radioactive **^{99m}Tc-BBN** complexes (Red trace; γ detection; Rt = 17. 8 min); **(B)** HPLC chromatogram (method 1) of Pz-BBN ligand (dark blue trace; UV detection 220 nm; Rt = 16.0 min).

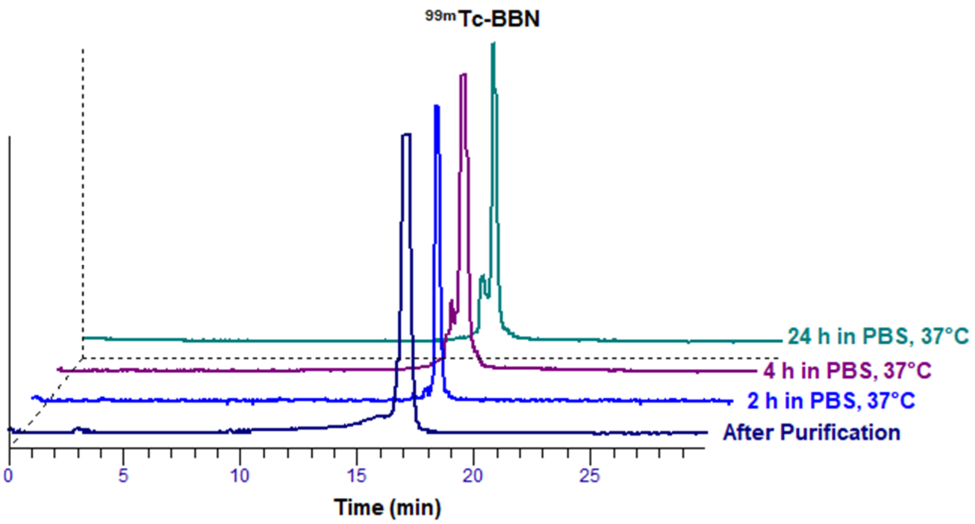

Figure S10. RP-HPLC radiochromatograms of **^{99m}Tc-BBN** after incubation in PBS pH 7.4 at 37 °C for 0 h, 2 h, 4 h and 24 h.

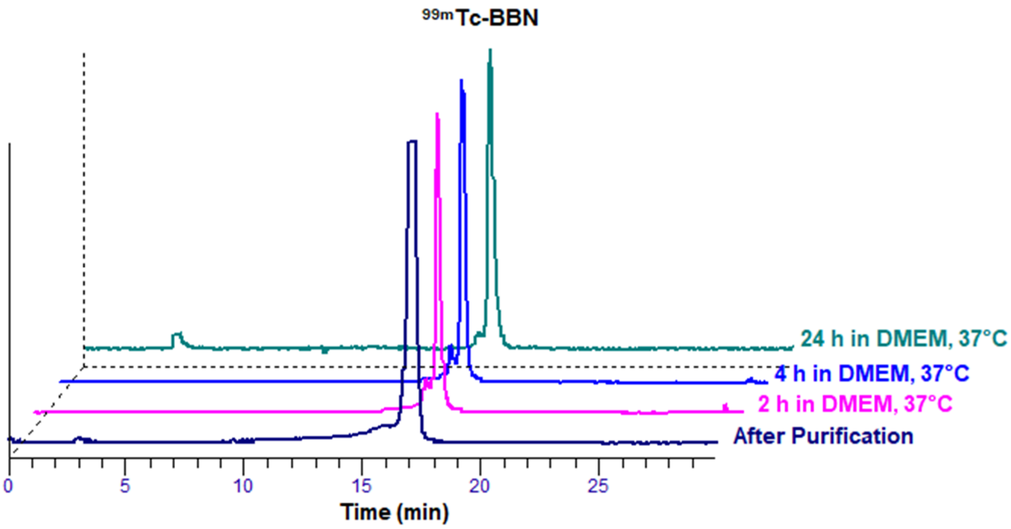

Figure S11. RP-HPLC radiochromatograms of **^{99m}Tc-BBN** after incubation in DMEM at 37 °C for 0 h, 2 h, 4 h and 24 h.

(A)

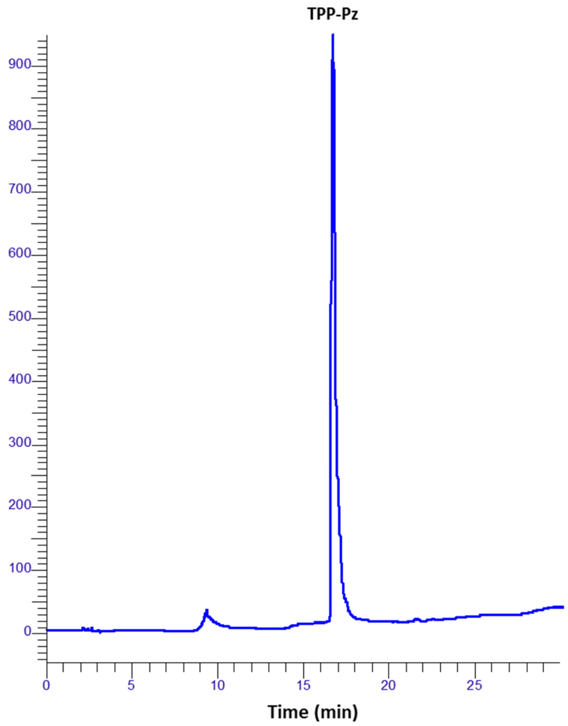

(B)

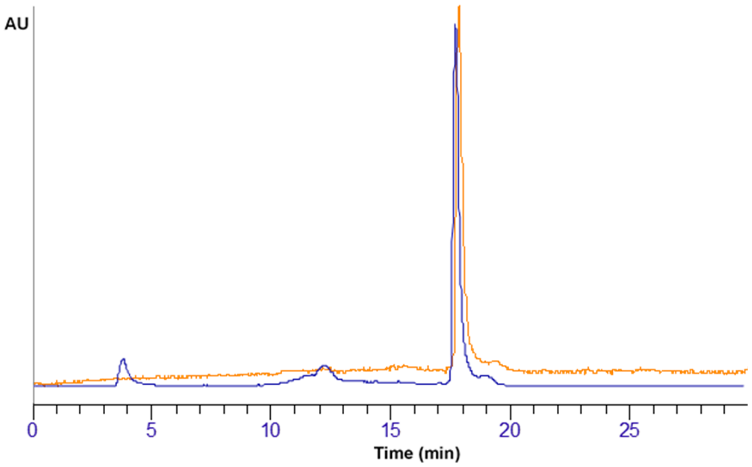

Figure S12. HPLC chromatograms (method 1): **(A)** TPP-Pz ligand (UV detection at λ=254 nm); **(B)** Re-TPP (Blue trace; UV detection 254 nm; Rt = 18.0 min) and radioactive ^{99m}Tc-TPP complexes (Orange trace; γ detection; Rt = 18.3 min).

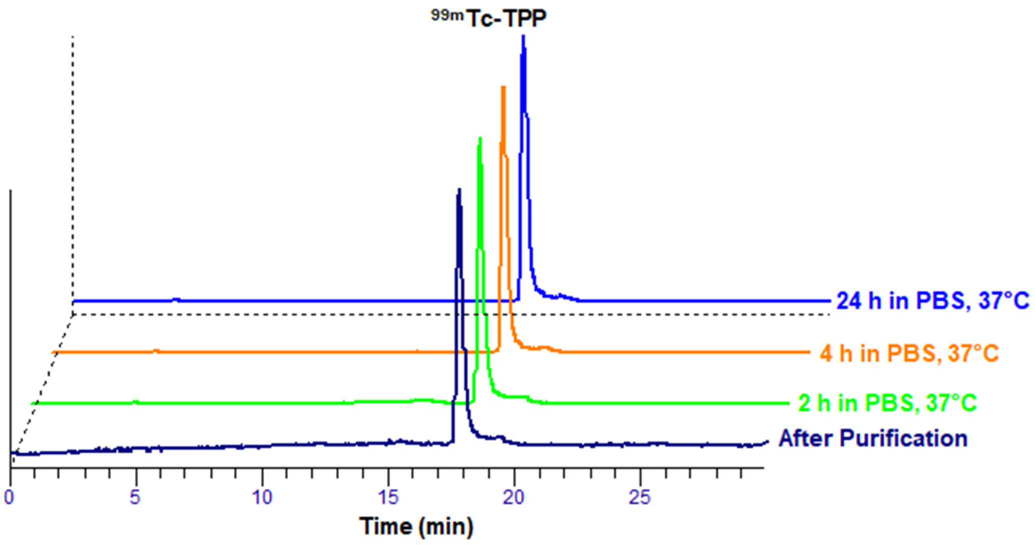

Figure S13. RP-HPLC radiochromatograms (method 1) of **^{99m}Tc-TPP** diluted in PBS pH 7.4 and incubation at 37 °C for 0 h, 2 h, 4 h and 24 h.

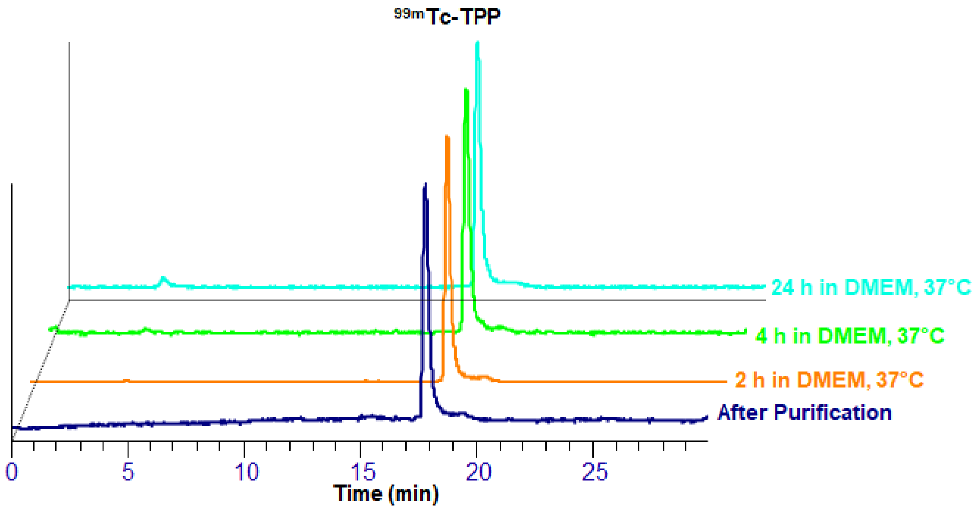

Figure S14. RP-HPLC radiochromatograms (method 1) of **^{99m}Tc-TPP** diluted in DMEM and incubation at 37 °C for 0 h, 2 h, 4 h and 24 h.

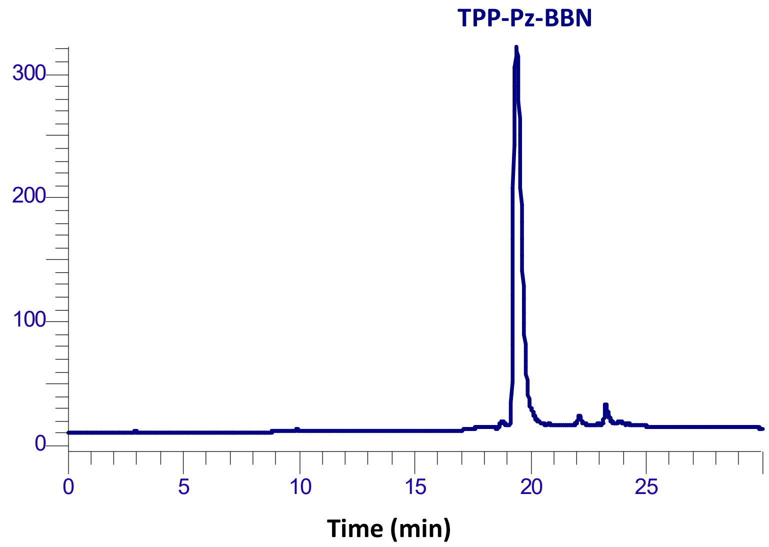

Figure S15. HPLC chromatograms (method 2) of TPP-Pz-BBN (detection at λ=254 nm);

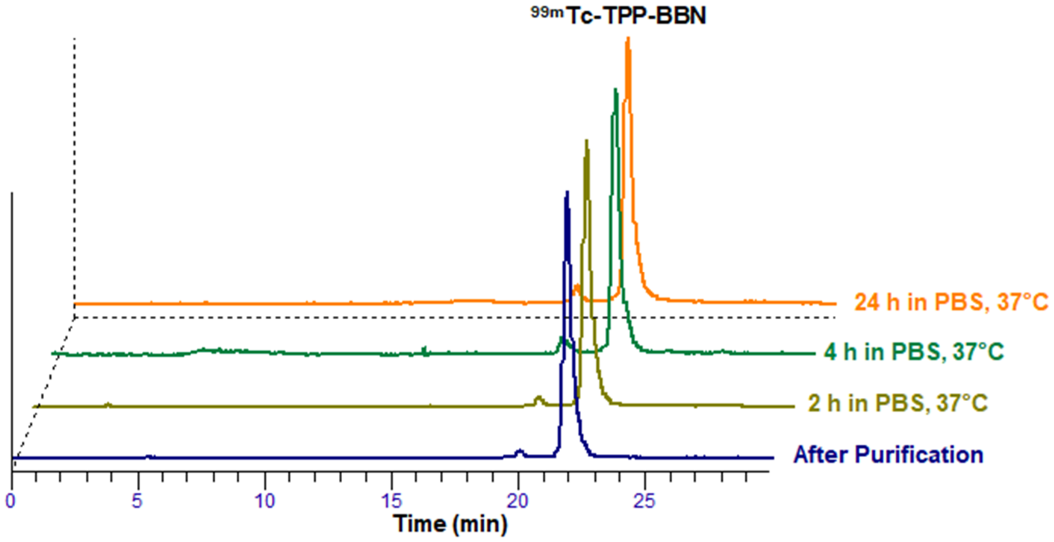

Figure S16. RP-HPLC radiochromatograms (method 2) of **^{99m}Tc-TPP-BBN** diluted in PBS pH 7.4 and incubation at 37 °C for 0 h, 2 h, 4 h and 24 h.

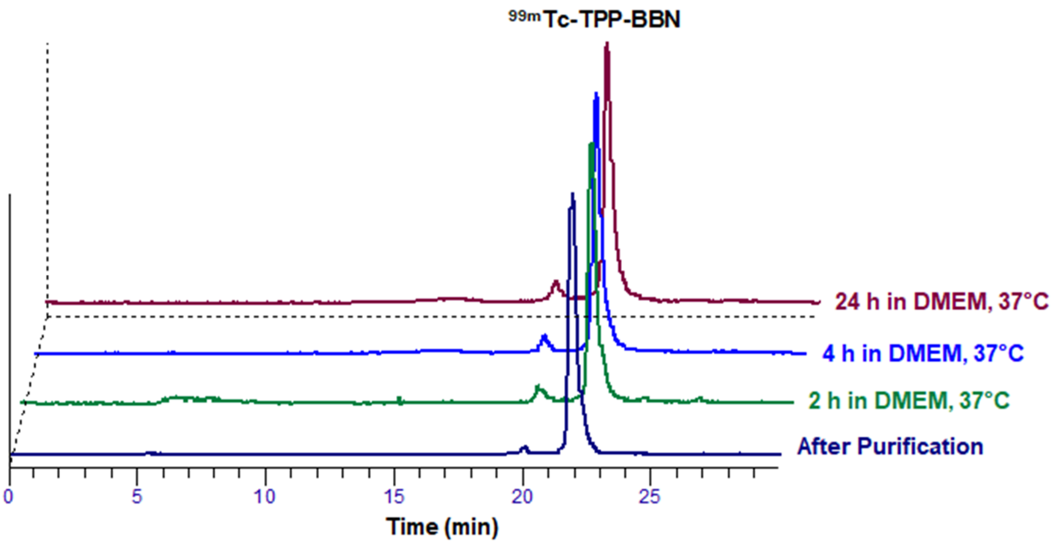

Figure S17. RP-HPLC radiochromatograms (method 2) of **^{99m}Tc-TPP-BBN** diluted in DMEM and incubation at 37 °C for 0 h, 2 h, 4 h and 24 h.

IV- Biological Evaluation: enzymatic assays and cell studies

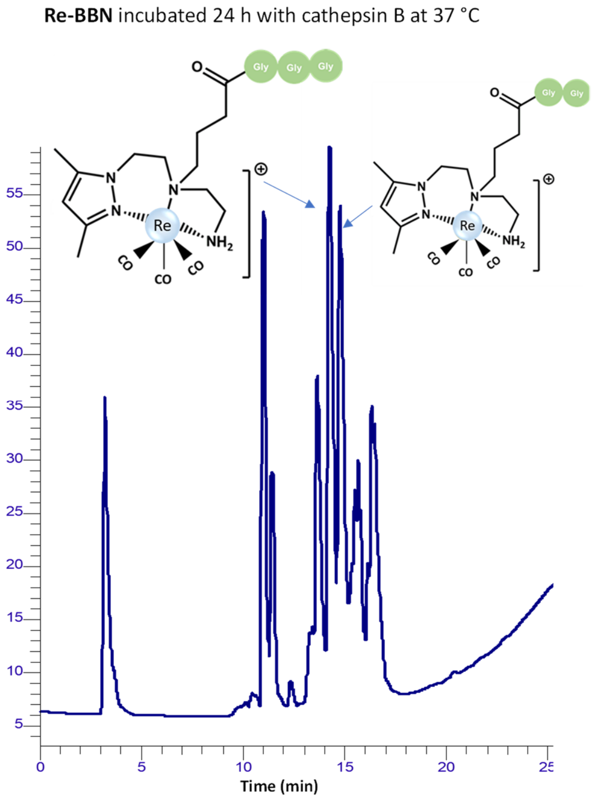

Figure S18. RP-HPLC chromatogram of the reaction mixture obtained after incubation of **Re-BBN** (λ = 254 nm) with cathepsin B for 24 h at 37 °C. The peaks were collected and identified by ESI-MS analysis.

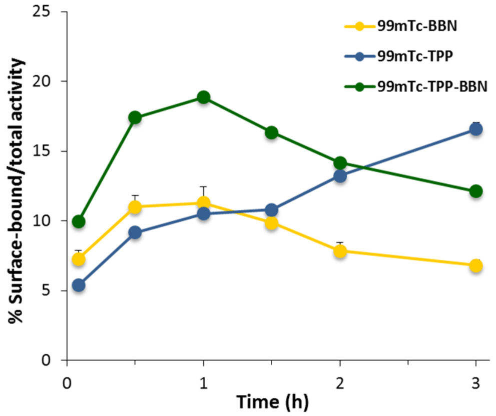

Figure S19. Time-dependent surface-bound of ^{99m}Tc-TPP-BBN (green), ^{99m}Tc-TPP (blue) and ^{99m}Tc-BBN (yellow) in PC3 cells at 37 °C. Results were expressed as a percentage of the total (applied) activity. Results were calculated from independent biological replicates (n = 4), and are given as the average ± SEM.

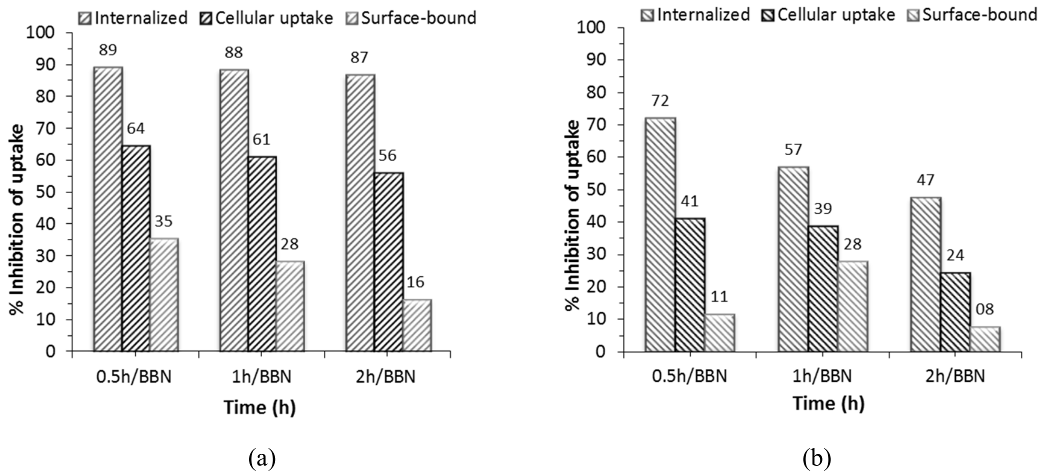

Figure S20. GRP receptor-blocking study: inhibition of cellular uptake, surface-bound and internalization of ^{99m}Tc-TPP-BBN (a) and ^{99m}Tc-BBN (b) by coincubation with [Tyr^{4}]-BBN (0.25 µg/0.5 mL/well) in PC3 cells at 37 °C. Data was expressed as a % of inhibition.
